# Isoselective Polymerization of 1‐Vinylcyclohexene (VCH) and a Terpene Derived Monomer *S*‐4‐Isopropenyl‐1‐vinyl‐1‐cyclohexene (IVC), and Its Binary Copolymerization with Linear Terpenes

**DOI:** 10.1002/marc.202400834

**Published:** 2024-11-18

**Authors:** Ilaria Grimaldi, Assunta D'Amato, Mariarosaria C. Gambardella, Antonio Buonerba, Raffaele Marzocchi, Finizia Auriemma, Carmine Capacchione

**Affiliations:** ^1^ Dipartimento di Chimica e Biologia “Adolfo Zambelli” Università degli Studi di Salerno, Via Giovanni Paolo II Fisciano SA 84084 Italy; ^2^ Dipartimento di Scienze Chimiche Università di Napoli Federico II Complesso Monte S. Angelo Via Cintia Napoli 80126 Italy

**Keywords:** catalyst performance, isotactic polymers, [OSSO]‐type titanium complexes, stereoregular polymerization, terpene‐based monomers

## Abstract

The advancement of stereoregular polymerization techniques for linear 1,3‐dienes has enabled the production of polymers with precise stereocontrol, influencing their physical and chemical properties significantly. While 1,3‐butadiene and isoprene yield diverse stereoregular polymers, cyclic dienes have received less attention due to catalyst challenges and limited application in the rubber industry. However, the growing interest in bio‐based monomers, particularly those derived from terpenes and terpenoids, has revitalized interest in cyclic monomers with conjugated double bonds. This study investigates 1‐vinylcyclohexene (VCH) polymerization using [OSSO]‐type titanium complexes **1**–**2**, revealing significant regio‐ and stereoselectivity. Catalyst **2**, incorporating cumyl substituents, demonstrates superior performance, yielding highly isotactic poly(VCH) with 3,4‐insertion predominance. It is also shown that the polymerization of *S*‐4‐isopropenyl‐1‐vinyl‐1‐cyclohexene (IVC), a bio‐based monomer, results in a highly isotactic polymer. Finally, the copolymerization results of IVC with two linear terpenes to obtain copolymers derived entirely from renewable sources are also reported.

## Graphical abstract

*Iso‐ and regio‐ specific advances*: The highly regio‐ and stereoselective polymerization of 1‐vinylcyclohexene (VCH) using [OSSO]‐type titanium complexes has been achieved. The catalyst bearing cumyl substituents demonstrates superior performance, yielding poly(VCH) with highly isotactic 3,4‐insertion of the monomer. In addition, the polymerization of *S*‐4‐isopropenyl‐1‐vinyl‐1‐cyclohexene, a bio‐based monomer, also afforded a highly isotactic and regioregular polymer and copolymers when copolymerized with linear terpenes.

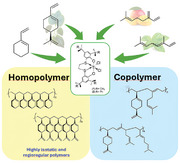

## 1 Introduction

The stereoregular polymerization of linear 1,3‐dienes has reached a high degree of sophistication with the possibility to obtain polymers with a complete control of the stereochemistry, thus determining the physical and chemical properties of the final polymeric material.^[^ ^1^, ^2^ ^]^ Indeed, monomers like 1,3‐butadiene or isoprene can be polymerized, giving a complete portfolio of stereoregular polymers (1,2 or 3,4 iso‐ and syndiotactic, 1,4‐*cis* or *trans*). Conversely, cyclic dienes have received limited attention due to the difficulties in finding efficient and selective catalysts and for the scarce appeal, the rubber industry, of the final polymeric products.^[^ ^3^, ^4^, ^5^ ^]^ More recently, the growing interest in bio‐based monomers has sparked a renewed impulse to the polymerization of cyclic monomers containing conjugated double bonds.^[^ ^6^, ^7^ ^]^ In particular, cyclic structural motifs are ubiquitous in natural products such as terpenes and terpenoids that can be directly polymerized or easily converted into polymerizable 1,3‐dienes.^[^ ^8^, ^9^, ^10^, ^11^ ^]^ As elegantly shown by Frey and coworkers, 1‐vinylcyclohexene (VCH) represents an ideal model molecule for studying cyclic conjugated diene anionic polymerization behavior.^[^ ^12^ ^]^ Notably, to date, there are very few reports on the polymerization of VCH,^[^ ^13^, ^14^ ^]^ and in only one case, the isospecific polymerization was achieved, albeit with poor activity, not allowing the complete characterization of the resulting polymer.^[^ ^15^ ^]^ Among the homogeneous catalysts that can be viable candidate for the stereoselective polymerization of VCH, [OSSO]‐type titanium complexes, when activated by methylalumoxane (MAO), have shown excellent performances in terms of activity and stereocontrol in the polymerization of styrene and conjugated dienes.^[^ ^16^, ^17^, ^18^, ^19^ ^]^

**Scheme 1:**
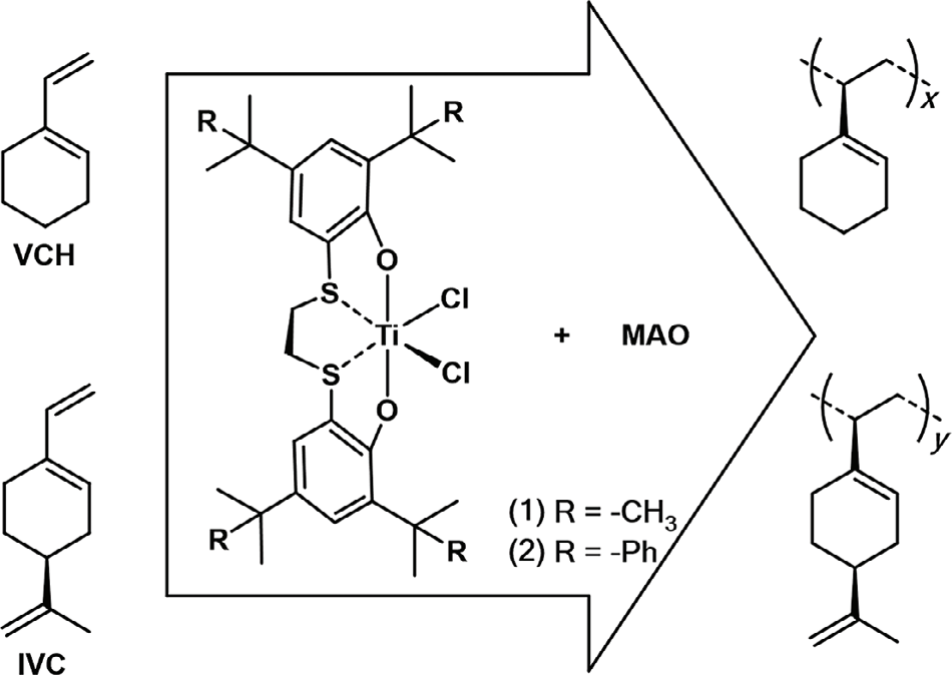
Isospecific polymerization of VCH and IVC promoted by [OSSO]titanium complexes (**1**) and (**2**) activated with MAO.

Indeed, due to the structural similarity of VCH and styrene, we decided to test these catalytic systems in the polymerization of VCH and a terpene‐based monomer with a similar structural motif: *S*‐4‐isopropenyl‐1‐vinyl‐1‐cyclohexene (IVC) (**Scheme** **1**).

## 2 Results and Discussion

The performance in the stereoselective polymerization of VCH of the two titanium complexes **1** and **2** (Scheme 1), bearing [OSSO]‐type ligand, activated by MAO, was compared, and the results are reported in **Table** **1**. Both these [OSSO]‐type Ti catalysts were found to be active in producing polymers with predominantly 3,4‐insertion of VCH along the polymer chain.

**Table 1:** Homopolymerization of VCH and IVC in the presence of catalysts 1–2 activated with MAO.

Run^a)^	Monomer	Cat	*T* [°C]	Time [h]	Yield [%]	3,4‐^b)^ [%]	*mmmm* ^b)^ [%]	M_n_ ^c)^ [kg mol^−1^]	Đ^c)^	*T*_g_ ^d)^ [°C]
1	VCH	1	25	18	17	80	n.d.	15.7	1.5	79.9
2	VCH	1	40	18	39	75	n.d.	28.2	1.7	80.8
3	VCH	2	0	18	4	>99	>99	13.5	1.9	86.6
4	VCH	2	25	18	54	>99	>99	104.2	2.2	96.0
5	VCH	2	40	18	59	>99	>99	125.7	2.0	95.7
6	VCH	2	80	18	57	>99	>99	84.8	2.0	92.1
7	VCH	2	40	5	53	>99	>99	134.4	1.9	96.7
8^e)^	VCH	2	40	18	82	>99	>99	103.8	1.9	98.9
9^f)^	IVC	2	25	18	32	>99	>99	34.3	2.1	81.8
10^f)^	IVC	2	40	18	80	>99	>99	158.4	1.4	74.9
11^f)^	IVC	2	80	18	90	>99	>99	124.0	1.6	78.2
12^f^ ^)^	IVC	2	80	5	93	>99	>99	124.2	1.7	74.9

^a)^ Reaction conditions: Catalyst (1.0 × 10^−5^ mol), [Al]/[Ti] = 500, VCH (3 mmol, 0.3 g), toluene (3 mL);

^b)^ Determined by ^1^H and ^13^C NMR spectroscopy;

^c)^ Determined by GPC;

^d)^ Determined by DSC;

^e)^ Catalyst (5.0 × 10^−5^ mol), [Al]/[Ti] = 500, VCH (14 mmol, 1.5 g), toluene (15 mL);

^f)^ Catalyst (1.0 × 10^−5^ mol), [Al]/[Ti] = 500, IVC (2 mmol, 0.3 g), toluene (3 mL).

Catalyst **2**, due to its greater steric hindrance, exhibited significantly higher regio‐ and stereoselectivity when compared to catalyst **1** (see runs 2 and 5, Table 1). Specifically, at a temperature of 40 °C, poly(vinylcyclohexene) (PVCH) was obtained with a higher yield, showing 99% of 3,4‐insertion and high isotacticity with *mmmm* >99%, as evidenced by ^13^C and ^1^H NMR (see Figures S6 and S8, Supporting Information).

The control of molecular weights is consistent with the behavior shown by [OSSO]‐type complexes as a single‐site catalyst in the polymerization of diene monomers as evidenced by the value of the dispersity (*Đ*) obtained from gel permeation chromatography (GPC) analyses.^[^ ^17^, ^18^, ^19^ ^]^

**Figure 1:**
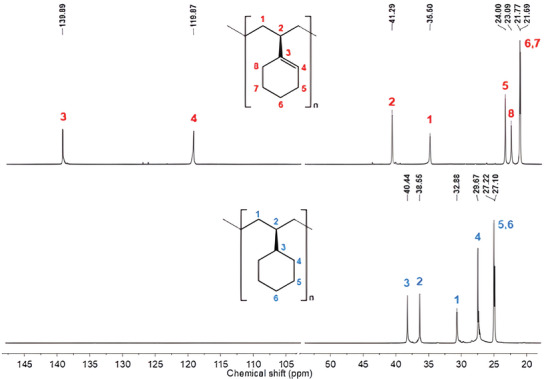
^13^C NMR spectra (150 MHz, CDCl_3_, 298 K) of isotactic poly(vinylcyclohexene) (sample **8**, Table 1) before (a) and after hydrogenation to isotactic poly(vinylcyclohexane) (b).

**Figure 2:**
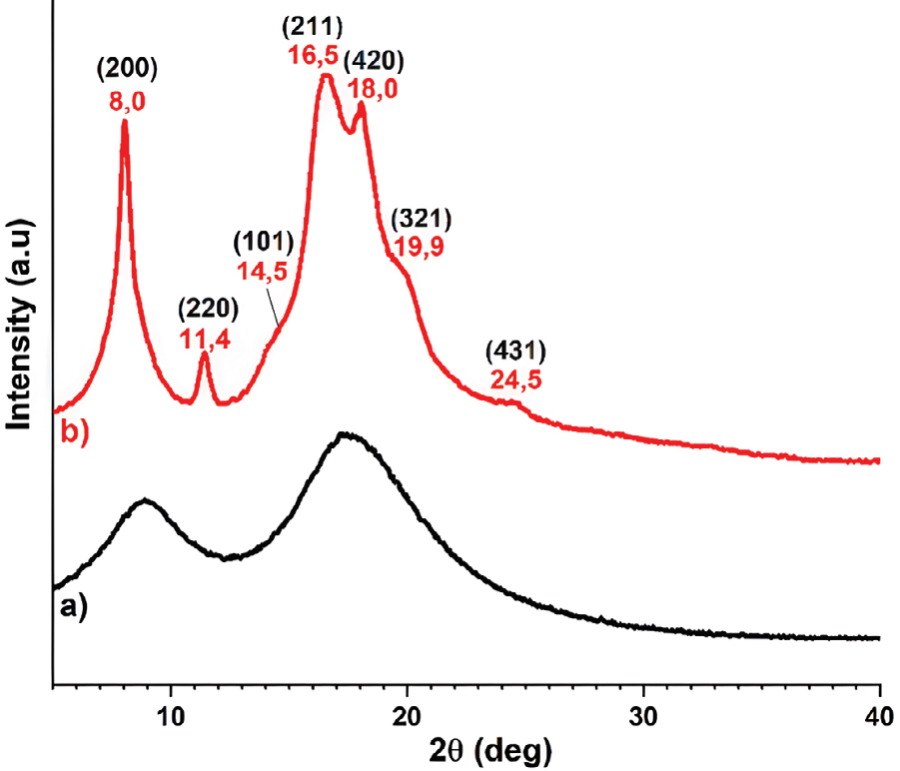
WAXS profiles of isotactic poly(vinylcyclohexene) before (black curve) and after hydrogenation to isotactic poly(vinylcyclohexane) (red curve).

The glass transition (*T*_g_) values were sensibly higher than those reported for the polymer obtained from anionic polymerization (80–99 °C),^[^ ^12^ ^]^ but despite the high degree of stereoregularity, no melting point was observed in the thermal analysis. As further proof of the high stereoregularity of the obtained polymer, the exhaustive hydrogenation of the sample obtained in run 8 led to the formation of semicrystalline isotactic poly(vinylcyclohexane) as confirmed by NMR and WAXS analyses (**Figures** **1** and **2**, vide infra). It is worth noting that isotactic PVCH, being the “missing link” between two semicrystalline isotactic polymers such as isotactic polystyrene and isotactic poly(vinylcyclohexane), is amorphous in nature.

The microstructure of the synthesized PVCH was determined by NMR analysis (see Figures 1 and S3–S7, Supporting Information). The ^13^C NMR signals of the PVCH (Figure 1a) indicate the formation of a highly isotactic polymer. The diagnostic chemical shifts of carbon 1 (35.5 ppm) and carbon 2 (41.29 ppm), highly sensitive to the tacticity of the polymer, are compatible with the previously reported chemical shifts for isotactic PVCH.^[^ ^15^ ^]^ Considering the presence of a single ^13^C signal for the carbon 2 of the PVCH, it can be concluded that the polymer has high isotacticity (i.e., *mmmm* > 99%). As a further proof of the isotactic nature of the synthesized polymer, the ^13^C NMR signals of hydrogenated PVCH (curve b of Figure 1), i.e., for the resulting poly(vinylcyclohexane), are fully in agreement with those of hydrogenated isotactic polystyrene.^[^ ^20^ ^]^

It is worth noting that, in spite of the high stereoregularity, the as‐synthesized PVCH samples are not crystalline (see, for instance, the WAXS curve a of Figure 2), and it is unable to crystallize from the melt, even after prolonged (at least 2 h) annealing at temperatures above the glass transition neither by solution using solvents such as toluene or chloroform. Possible reasons for the lack of crystallinity of PVCH may be envisaged in a too‐slow crystallization kinetic, in the adopted trial conditions. Therefore, isotactic PVCH represents a non‐common example of polymer not able to crystallize despite its regular constitution and configuration. Similar behavior is shown by enantiomerically pure species of isotactic poly(limonene carbonate) derived from citrus oil and CO_2_.^[^ ^21^, ^22^ ^]^

Owing to the isospecific polymerization of VCH using catalyst **2**, we also performed the polymerization of *S*‐4‐isopropenyl‐1‐vinyl‐1‐cyclohexene (IVC), a bio‐based monomer obtained by perillaldehyde.^[^ ^23^ ^]^ We conducted polymerizations at varying temperatures and observed a notable increase in yield as the temperature was raised. The most favorable outcome was achieved at 80 °C even in 5 hours (see run 12, Table 1). Also in this case, a highly isotactic polymer was obtained as the ^13^C and ^1^H NMR spectra confirm (see Figures S8 and S9, Supporting Information), as the highly isotactic microstructure in agreement with the polymer obtained by using lanthanide catalysts.^[^ ^23^ ^]^

The polymerization of IVC to poly(*S*‐4‐isopropenyl‐1‐vinyl‐1‐cyclohexene) (PIVC) was undertaken to explore the potential of creating novel materials with unique properties that could complement or enhance those of PVCH. By examining both IVC and VCH, we aimed to understand the influence of structural variations on polymer properties, particularly in relation to thermal behavior and crystallinity. In addition, it is important to note that PIVC is a polymer derived from renewable sources, which makes it a sustainable alternative in polymer chemistry. Isotactic PIVC exhibits distinct glass transition temperatures (*T*_g_) compared to PVCH (see Figures S16–S21, Supporting Information). Reasonably, the presence of the isopropenyl group in PIVC results in different chain dynamics and packing efficiency, influencing *T*_g_. However, as shown by WAXS analysis (Figure S33, Supporting Information), like PVCH, PIVC is amorphous and unable to crystallize under the common crystallization conditions, even after prolonged annealing at temperatures close to *T*_g_.

While it is generally expected that the *T*_g_ increases with molecular weight due to reduced chain mobility, this relationship is not always straightforward.^[^ ^24^ ^]^ Several factors can influence *T*_g_ beyond just molecular weight like architecture of the polymer, intermolecular interactions, polymer crystallinity, and so on. Given these complexities, it is not surprising that some high molecular weight samples exhibit lower *T*_g_ values.^[^ ^25^, ^26^ ^]^

**Figure 3:**
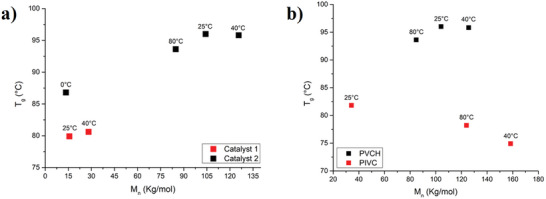
Diagrams showing the correlation between molecular weight (*M*_n_) and glass transition temperature (*T*_g_): a) comparison of PVCH samples synthesized using catalysts **1** and **2**; b) comparison between PVCH and PIVC samples synthesized with catalyst **2**. The polymerization temperatures are indicated.

In **Figure** **3**a, we report the diagram relating molecular weights to glass transition temperatures for PVCH samples. From the comparison between the two catalysts, it is evident that under identical conditions, the molecular weights are significantly different. Higher regioselectivity correlates with a higher *T*_g_ value of the polymer. Catalyst **1**, with lower regioselectivity and stereoselectivity, results in lower molecular weights and, consequently, lower *T*_g_ values. For instance, at polymerization temperature of 40 °C, the *T*_g_ value increases from 80.8 to 95.8 °C as the 3,4‐selectivity of the polymer obtained from catalyst **2** increased from 75% to over 99% (see runs 2 and 5, Table 1). Instead, in Figure 3b, we present a comparison between PVCH and PIVC samples synthesized with catalyst **2** at various temperatures. For PVCH, *T*_g_ increases as molecular weight rises, whereas for PIVC, the opposite trend is observed. Our study found that the *T*_g_ of isotactic PIVC is lower than that of PVCH, indicating a more flexible polymer structure due to the bulkier side groups disrupting efficient chain packing.

PVCH and PVIC are both fragile. As an example, the stress–strain curves of films cast from toluene solutions of PVCH (Entry 8 of Table 1) and PVIC (Entry 11 of Table 1) are shown in Figure S34 (Supporting Information). Both samples are fragile, with values of deformation at break ε_b_ less than 8 and 2%, respectively, and rigid, with values of Young's modulus of ≈25 and 1090 MPa, respectively.

**Scheme 2:**
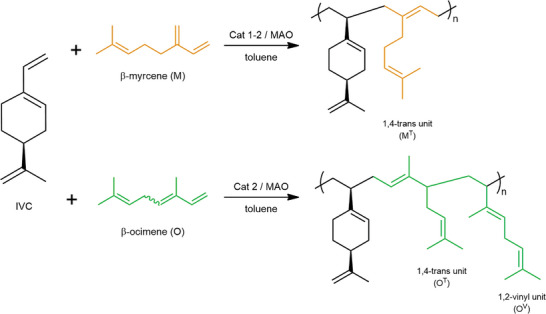
Copolymerization of IVC and linear terpenes promoted by [OSSO]titanium complexes (**1**) and (**2**) activated with MAO.

To extend the study to the synthesis of new polymers from renewable sources, the IVC was copolymerized with two linear terpenes, β‐myrcene and β‐ocimene (**Scheme** **2**). The copolymerization reactions were carried out under controlled conditions to ensure the efficient incorporation of the terpene monomers into the polymer backbone.

The resulting copolymers were characterized using various techniques, including NMR spectroscopy, GPC, and differential scanning calorimetry (DSC). These analyses provided insights into the composition, molecular weight distribution, and thermal properties of the copolymers. The results, summarized in **Table** **2**, demonstrate the successful synthesis of IVC‐based copolymers with different properties, highlighting the potential of β‐myrcene and β‐ocimene as comonomers in the development of sustainable polymeric materials.

**Table 2:** Copolymerization of IVC and linear terpenes in presence of catalysts 1–2/MAO.

Run ^a^ ^)^	Terpene/feed [mol%]	Cat	Yield [%]	*M*_n_ ^b)^ [kg mol^−1^]	Đ^b)^	Polymer composition^c)^ [%]		*T*_g_ ^d)^ [°C]
						Terp. [mol%]	IVC [mol%]	
1	M / 50	1	35	28.1	2.0	35.6	64.4	33.6
2	M / 80	1	17	12.3	2.6	73.9	26.1	−30.2
3	M / 50	2	40	18.8	2.1	24.3	75.7	36.3
4	M / 80	2	16	13.5	3.9	58.1	41.9	−30.2
5	O / 50	2	78	53.3	3.1	54.8	45.2	27.4
6	O / 80	2	52	40.4	2.6	79.4	20.6	3.4

^a)^ Reaction conditions: Catalyst (5.0 × 10^−6^ mol), ([IVC]+[Terp])/[Ti] = 1000, [Al]/[Ti] = 500, toluene (5 mL), 80 °C, 24 h;

^b)^ Determined by GPC;

^c)^ Determined by ^1^H and ^13^C NMR spectroscopy;

^d)^ Determined by DSC.

Several one‐pot copolymerizations of IVC with both linear terpenes were conducted, varying the molar ratios of the monomers. For the copolymerizations of IVC with β‐myrcene, a comparison between catalyst **1** and catalyst **2**, both activated by methylaluminoxane (MAO), was performed. The results indicate that, although yields are similar, catalyst **1** results in a higher incorporation of myrcene in the final polymer, for example, from 24 to 36 mol% (see runs 1 and 3, Table 2). This finding aligns with previous results,^[^ ^27^, ^28^ ^]^ which showed that catalyst **1** had superior performance in the homopolymerization of β‐myrcene, while catalyst **2** was more efficient for β‐ocimene. Indeed, in the copolymerization with β‐ocimene, catalyst **2** was employed, resulting in the formation of copolymers that closely match the molar ratios of the two monomers used. The copolymers were characterized using ^1^H NMR and ^13^C NMR (see Figures S10–S13, Supporting Information) to reveal their microstructure. The results indicate that with β‐myrcene, the copolymer predominantly exhibits 1,4‐*trans* insertion (M^T^); with β‐ocimene, as expected, both 1,4‐*trans* (O^T^) and 1,2‐vinyl (O^V^) insertions were observed. GPC analyses (see Figures S30–S32, Supporting Information) showed monomodal profiles, and the Đ values were consistent with their copolymeric nature. In addition, molecular weights and yields decreased with increasing terpene content in the polymer. DSC curves (see Figures S22 and S23, Supporting Information) of the copolymers revealed the presence of a single glass transition temperature (*T*_g_), which significantly decreased as the terpene content increased, indicating significant effects on the polymer characteristics.

## 3 Conclusion

In summary, in this study, isotactic PVCH has been successfully synthesized for the first time using titanium complexes bearing OSSO‐type ligands (complexes **1**–**2** in Scheme 1). Notably, complex **2**, bearing cumyl substituents, demonstrated superior activity and isospecificity compared to complex **1**, which bears *tert*‐butyl substituents, thus yielding a polymer with exceptionally high isotacticity (*mmmm* > 99%). Furthermore, it is worth noting that following the reduction of the endocyclic double bond, the polymer morphology changes from amorphous to semicrystalline, as can be seen from the DSC thermogram and the WAXS spectrum. In advancing sustainable chemistry, catalyst **2**, identified as the most effective, was further applied in the stereoselective polymerization of IVC, again affording a polymer exhibiting remarkable isotacticity. In addition, IVC was successfully copolymerized with two linear terpenes, β‐myrcene and β‐ocimene. The results showed that the polymer properties change as the terpene content in the chain increases allowing the synthesis of materials having a wide range of properties. In conclusion, our findings not only contribute to expanding the repertoire of polymer synthesis methodologies but also highlight the potential of this family of catalysts for further advancements in the field of sustainable, stereoregular polymeric materials.

## 4 Experimental Section

### General Information

Reagents and solvents were purchased from Sigma‐Aldrich, Merck, or TCI chemicals. Solvents were dried and distilled before use. All air‐ and/or water‐sensitive compound manipulations were carried out using a glovebox or standard Schlenk techniques under an N_2_ atmosphere. Commercial grade toluene (Sigma‐Aldrich) was dried over calcium chloride, refluxed 48 h under a nitrogen atmosphere over sodium and distilled before use. MAO (10 wt% solution in toluene; Sigma‐Aldrich) was used as received. Dichloro{1,4‐dithiabutanediyl‐2,20‐bis(4,6‐di‐alkylphenoxy)} titanium complexes (**1**) and (**2**) were synthesized according to the literature procedure.^[^ ^27^ ^]^ NMR spectra were recorded on a Bruker AM 300 spectrometer (300 MHz for ^1^H; 75 MHz for ^13^C), a Bruker AVANCE 400 spectrometer (400 MHz for ^1^H; 100 MHz for ^13^C), and a Bruker ASCEND 600 spectrometer (600 MHz for ^1^H; 150 MHz for ^13^C). ^1^H and ^13^C chemical shifts are listed in parts per million (ppm) referenced to tetramethylsilane (TMS) using the protio residual signal of the deuterated solvent. Spectra were reported as chemical shift (δ ppm), multiplicity, and integration. Multiplicity is abbreviated as follows: singlet (s), doublet (d), triplet (t), multiplet (m), broad (br), and overlapped (o). The number‐average molecular weights (*M*_n_) and molecular weight distributions of polymers (dispersity, *Ð*) were evaluated by GPC using an Agilent 1260 Infinity Series GPC chromatograph equipped with an RI, PLGPC 220 refractive index detector. All measurements were performed with THF as the eluent at a 1.0 mL min^−1^ flow rate at 35 °C. Monodisperse poly(styrene) polymers were used as calibration standards. DSC analyses were carried out with a Mettler Toledo DSC‐822 apparatus in a flowing N_2_ atmosphere at a rate of 10 °C min^−1^. Powder wide‐angle X‐ray diffraction (p‐WAXD) profiles were collected in reflection mode using a multipurpose PANalytical Empyrean diffractometer using the Ni‐filtered Cu Kα radiation (λ = 0.154 nm). Solution cast film were prepared by dissolving 100 mg of polymers in 15 mL of solvent (toluene or chloroform), by slow evaporation of solvent. Tensile properties were measured at room temperature using an Instron universal testing machine on rectangular specimens cut from toluene cast films with width, length, and thickness of 3, 10, and 0.5 mm, respectively. Stress–strain curves were measured according to the ASTM D638‐22 by stretching the specimens until failure using the ratio between the drawing speed and the specimen gauge length equal to 10 mm mm^−1^ min^−1^. The final curves were averaged over at least five independent experiments.

### Monomers Syntheses

VCH was synthesized according to the literature.^[^ ^12^ ^]^ 1‐Ethynylcyclohexene (13.5 mL) was selectively hydrogenated over Pd/CaCO_3_ (poisoned with lead, 1.0 g), at 1 bar of hydrogen for 8 h at room temperature in pentane (100 mL). The catalyst was then removed by filtration over celite, and the solvent was distilled under reduced pressure. The residue was purified by column chromatography on silica gel (100% *n*‐heptane), followed by anhydrification over CaH_2_, to obtain 1‐vinylcyclohexene in 72% yield. ^1^H NMR (600 MHz, CD_2_Cl_2_) δ: 6.35 (1H, dd, *J* 17.5, 10.7 Hz, *H* C═CH_2_), 5.77 (1H, br t, C═C *H*), 5.07 (1H, d, *J* 17.5 Hz, HC═C *H*_2_, *trans*), 4.88 (1H, d, *J* 10.7 Hz, HC═C *H*_2_, *cis*), 2.15–2.13 (4H, m, C *H 2* C═CHC *H 2*), 1.68 (2H, m, C═CHCH_2_C *H 2*), 1.61 (2H, m, C *H 2* CH_2_C═CH).

IVC was synthesized according to the literature.^[^ ^23^ ^]^ Potassium *tert*‐butoxide (7.4 g, 66.0 mmol) was added in portions to a suspension of methyltriphenylphosphonium bromide (21.4 g, 60.0 mmol) in dry THF (50 mL), and the reaction mixture was stirred at room temperature (RT) for 2 h. (*S*)‐(−)‐Perillaldehyde (7.5 g, 50.0 mmol) was then dropwise added into the above described reaction mixture at 0 °C. After stirring overnight at RT, the reaction was quenched by adding water, and the organics were extracted with diethyl ether. The organic solution was dried over MgSO_4_, and the solvent was subsequently removed under reduced pressure. The residue was treated with *n*‐hexane to precipitate triphenylphosphine oxide, and the solution was concentrated and purified by column chromatography on silica gel to give IVC with yield of 57%. ^1^H NMR (400 MHz, CDCl_3_, ppm): δ 6.38 (dd, J = 17.2, 10.8 Hz, 1H, CH_2_═C *H* ─C), 5.78 (s, 1H, C═C *H* ─CH_2_), 5.08 (d, J = 17.2 Hz, 1H, CH═C *H 2*), 4.92 (d, J = 10.8 Hz, 1H, CH═C *H 2*), 4.74 (s, 2H, C═C *H 2*), 2.19 (t, 5H, C─C *H 2* ─CH_2_+CH_2_─C *H* ─CH_2_+CH─C *H 2* ─CH), 1.92 (m, 1H, CH─C *H 2* ─CH_2_), 1.76 (s, 3H, C─C *H 3*), 1.52 (m, 1H, CH─C *H 2* ─CH_2_).

### Polymerization of 1‐Vinylcyclohexene (runs 1–8, Table 1)

The metal complex (complex **1** or **2**, 10 µmol) was added into a 10 mL Schlenk tube equipped with a magnetic stirrer and dissolved in 3 mL of dry toluene. MAO was added, and the solution was left stirring for 30 min to preactivate the metal complex. Then VCH (3 mmol, 0.3 g) was added, and the system was placed in a thermostated oil bath and stirred for 18 h. The polymers were coagulated in an excess of acidified methanol, washed several times with methanol, recovered by filtration, and dried in the vacuum oven overnight.

### Polymerization of *S*‐4‐Isopropenyl‐1‐vinyl‐1‐cyclohexene (runs 9–12, Table 1)

Complex **2** (10 µmol) was added into a 10 mL Schlenk tube equipped with a magnetic stirrer and dissolved in 3 mL of dry toluene. MAO was added, and the solution was left stirring for 30 min to preactivate the metal complex. Then IVC (2 mmol, 0.3 g) was added, and the system was placed in a thermostated oil bath at 80 °C and stirred for 18 h. The polymers were coagulated in an excess of acidified methanol, washed several times with methanol, recovered by filtration, and dried in the vacuum oven overnight.

### Hydrogenation of Poly(vinylcyclohexene) (run 8, Table 1)

Poly(vinylcyclohexene) was hydrogenated by dissolving polymer in 30 mL of *n*‐decane in an autoclave, thermostated at 130 °C in a sand bath, in the presence of 200 mg of palladium on charcoal and 30 bar of hydrogen. After 48 h, the mixture was filtered over celite and poured into ethanol. The polymer was recovered by filtration and dried in vacuum.

### Copolymerization of IVC with Linear Terpenes (runs 1–6, Table 2)

The metal complex (complex **1** or **2**, 5 µmol) was added into a 10 mL Schlenk tube equipped with a magnetic stirrer and dissolved in 4 mL of dry toluene. MAO was added, and the solution was left stirring for 30 min to preactivate the metal complex. Then both comonomers (IVC and myrcene/ocimene) were dissolved in 1 mL of dry toluene and added, and the system was placed in a thermostated oil bath at 80 °C and stirred for 24 h. The polymers were coagulated in an excess of acidified methanol, washed several times with methanol, recovered by filtration and dried in the vacuum oven overnight.

## Conflict of Interest

The authors declare no conflict of interest.

## Supporting information



Supporting Information

## Data Availability

The data that support the findings of this study are available from the corresponding author upon reasonable request.
