# Supplementary material for: Isoselective Polymerization of 1‐Vinylcyclohexene (VCH) and a Terpene Derived Monomer S‐4‐Isopropenyl‐1‐vinyl‐1‐cyclohexene (IVC), and Its Binary Copolymerization with Linear Terpenes
Source: Macromol Rapid Commun. 2024 Nov 18;46(5):2400834. doi: 10.1002/marc.202400834 (PMC11884221; doi:10.1002/marc.202400834)
Supplement: Supplementary file 1 — Supporting Information [file MARC-46-2400834-s001.docx]

**Isoselective Polymerization of 1-Vinylcyclohexene (VCH) and a Terpene Derived Monomer *S*-4-Isopropenyl-1-vinyl-1-cyclohexene (IVC), and Its Binary Copolymerization with Linear Terpenes**

Ilaria Grimaldi, Assunta D’Amato, Mariarosaria C. Gambardella, Antonio Buonerba, Raffaele Marzocchi^†^, Finizia Auriemma^†*^, and Carmine Capacchione^*^

Dipartimento di Chimica e Biologia “Adolfo Zambelli”, Università degli Studi di Salerno, Via Giovanni Paolo II, 84084 Fisciano, SA, Italy. E-mail: [ccapacchione@unisa.it](mailto:ccapacchione@unisa.it)

^†^Dipartimento di Scienze Chimiche Università di Napoli Federico II, Complesso Monte S. Angelo, Via Cintia, 80126, Napoli, Italy. E-mail: [auriemma@unina.it](mailto:auriemma@unina.it)

**SUPPORTING INFORMATION**

**Table of content**

[1. NMR characterization of the monomers 2](#_Toc181632001)

[2. NMR characterization of the polymers 3](#_Toc181632002)

[3. Hydrogenation of poly(vinylcyclohexene) 9](#_Toc181632003)

[4. DSC Thermal Analyses 10](#_Toc181632004)

[5. Thermogravimetric Analyses 14](#_Toc181632005)

[6. GPC Analyses 16](#_Toc181632006)

[7. Diffraction Analyses 19](#_Toc181632007)

[8. Stress-strain curves 19](#_Toc181632008)

[References 20](#_Toc181632009)

# **NMR characterization of the monomers**

**1-Vinylcyclohexene**

**
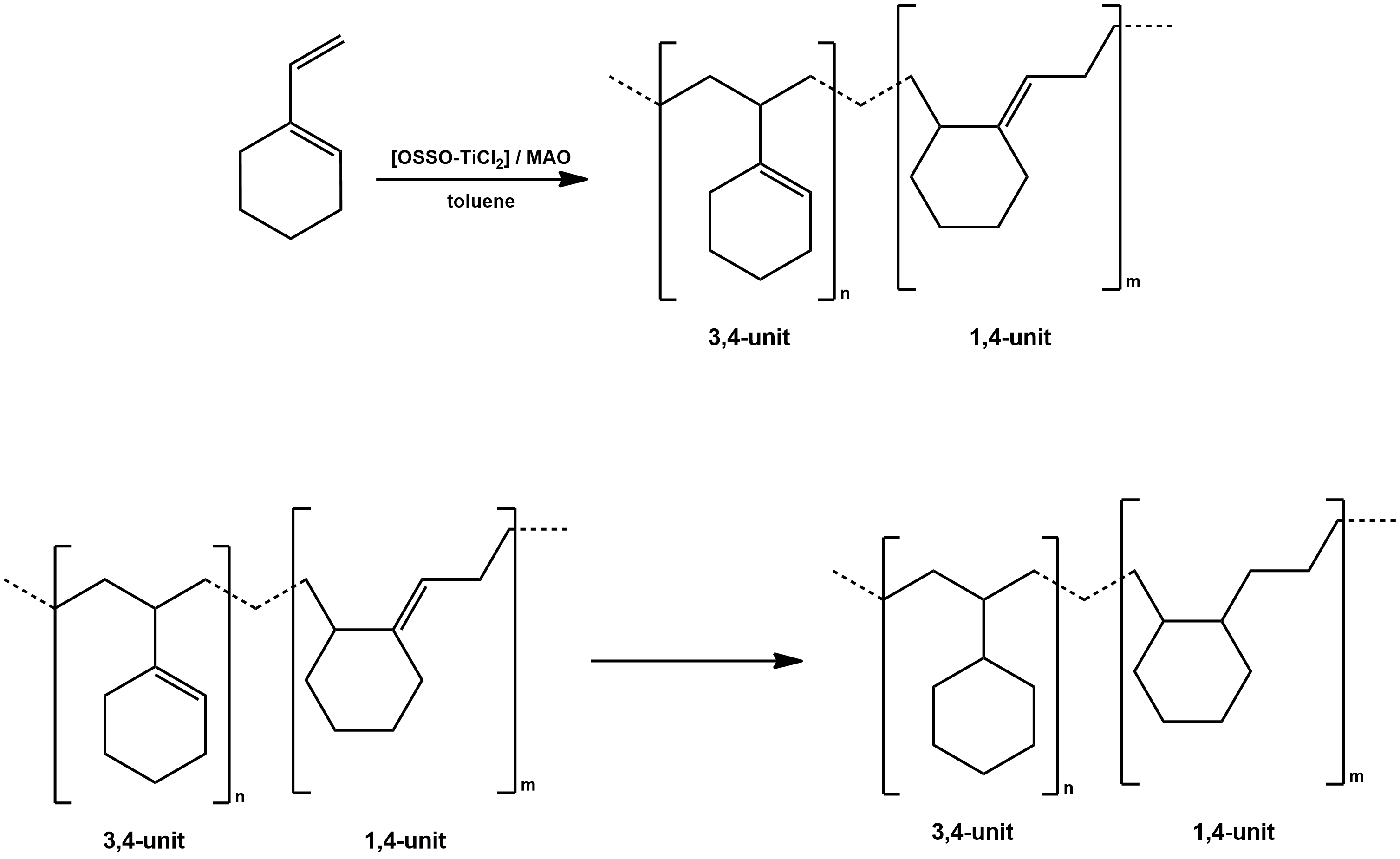
**
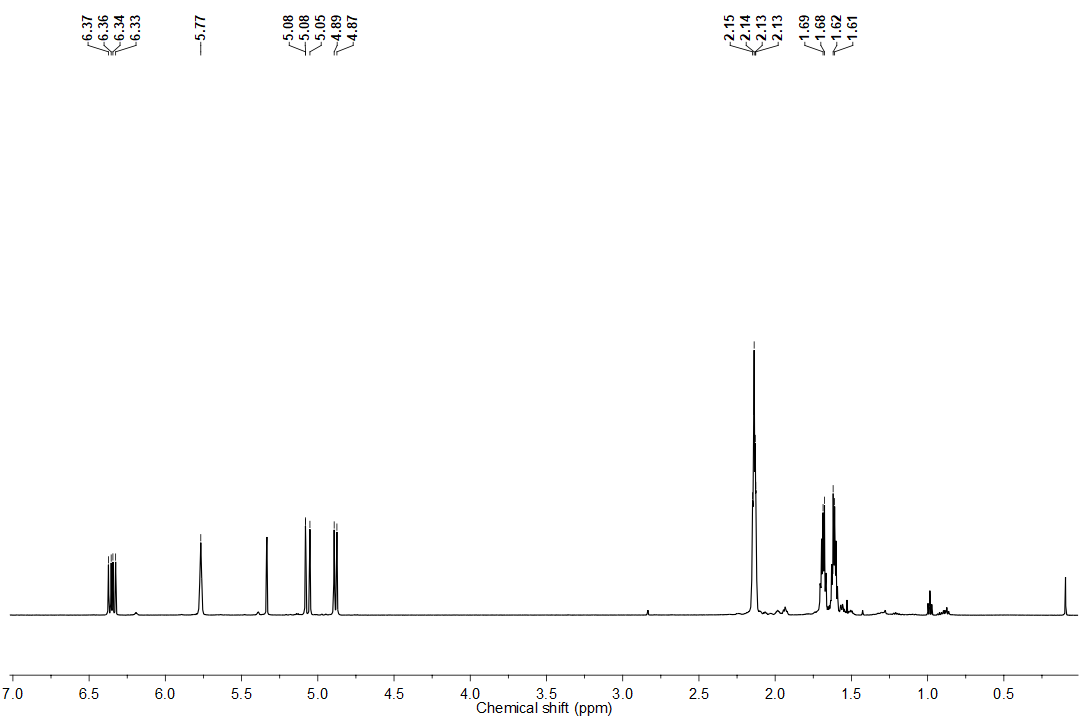


**Figure S1**. ^1^H NMR spectrum of 1-vinylcyclohexene (600 MHz, CD_2_Cl_2_, 298 K).

***S*-4-isopropenyl-1-vinyl-1-cyclohexene (IVC)**


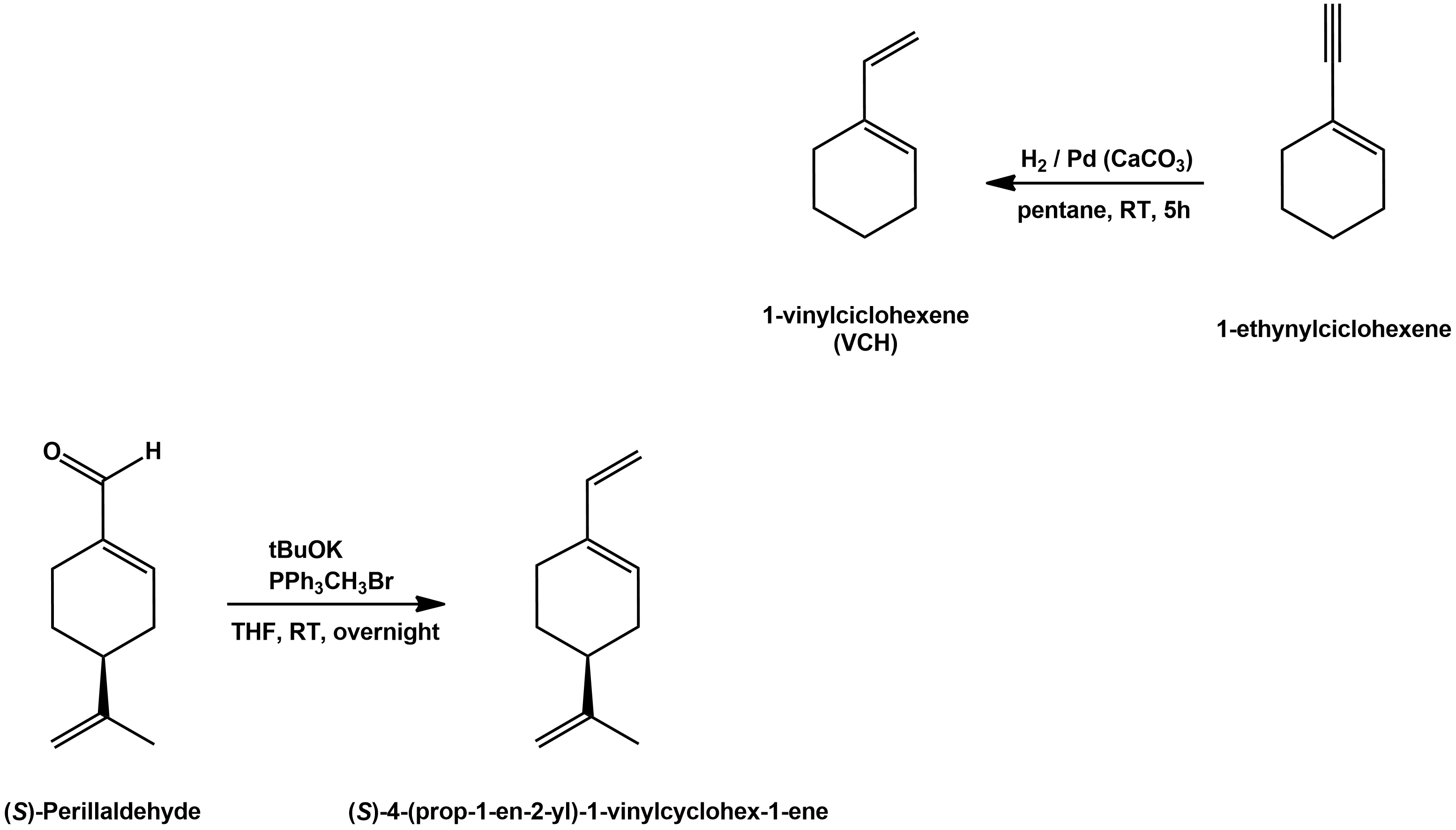

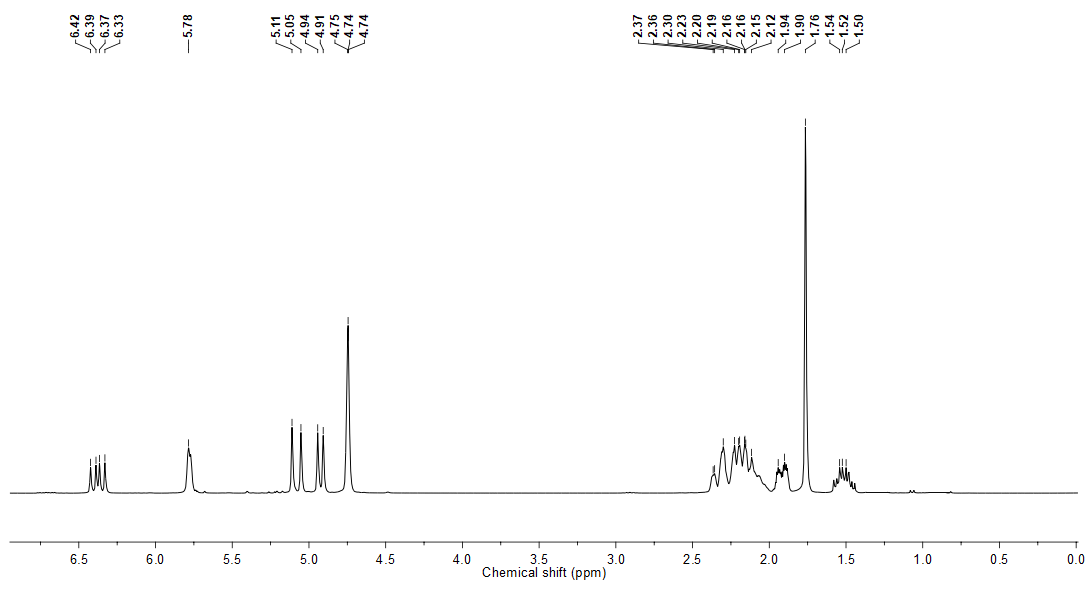


**Figure S2**. ^1^H NMR spectrum of *S*-4-isopropenyl-1-vinyl-1-cyclohexene (300 MHz, CDCl_3_, 298 K).

# **NMR characterization of the polymers**


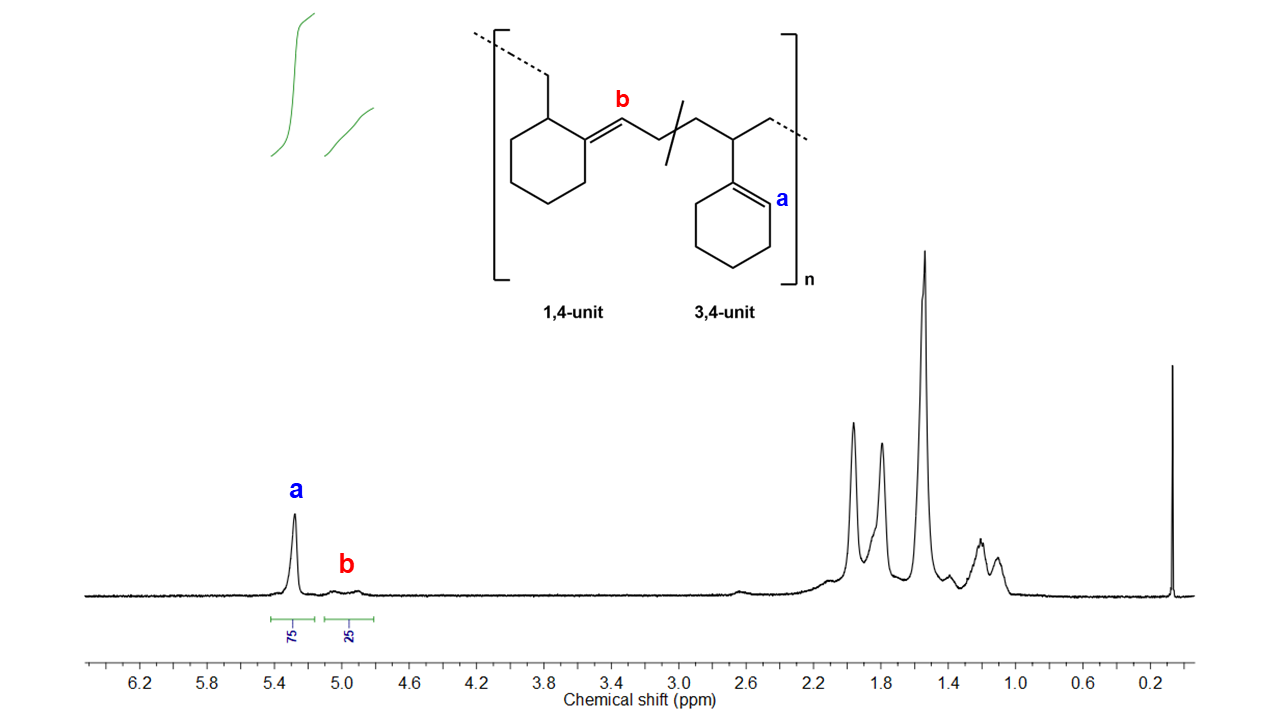


**Figure S3**. ^1^H NMR (400 MHz, CDCl_3_, 298 K) of entry **2**, Table 1. The peak at 5.29 ppm is related to the olefin proton of the 3.4 unit; the peak at 4.91 is related to the olefin proton of the 1,4-insertion.


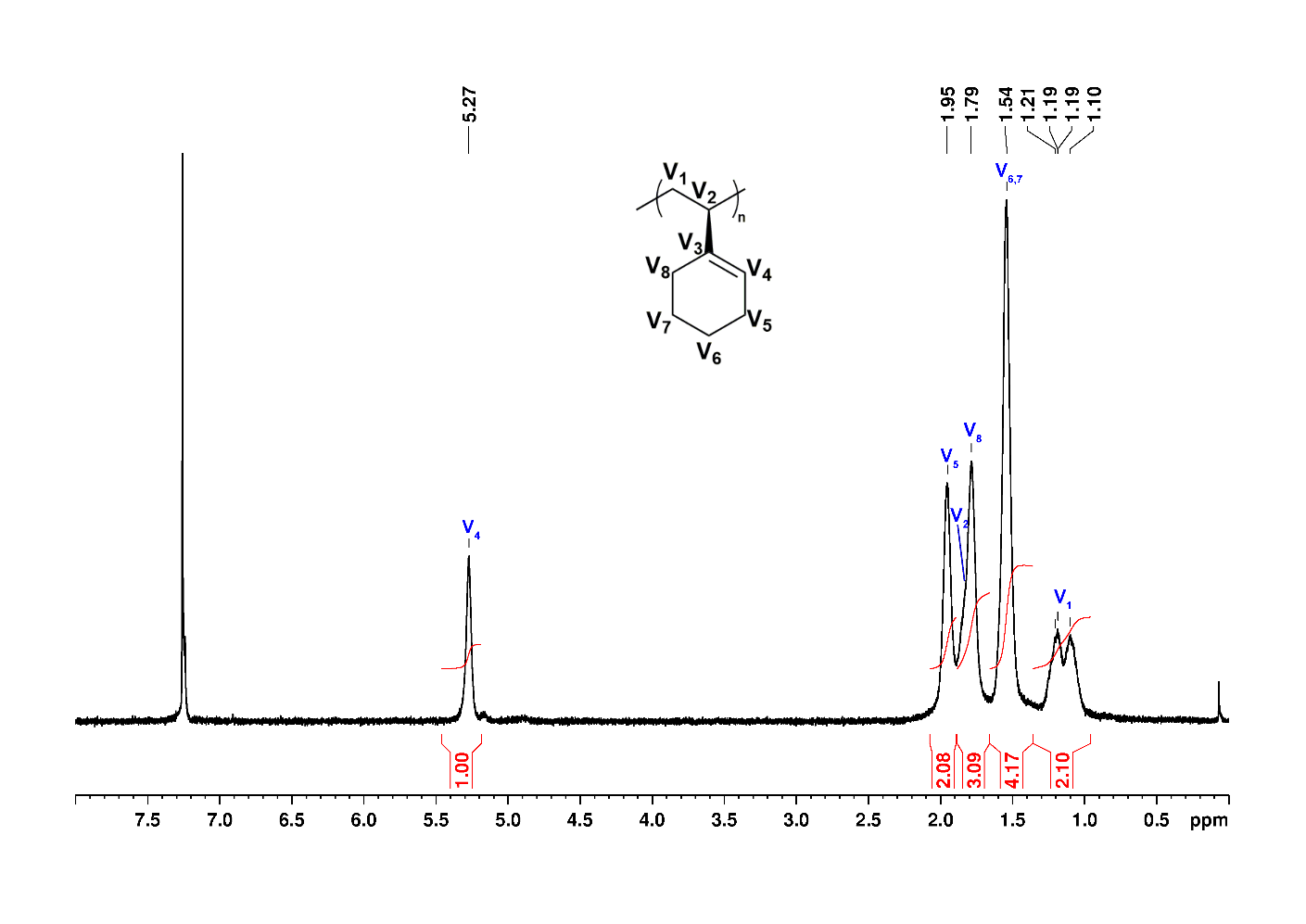


**Figure S4**.^1^H NMR (400 MHz, CDCl_3_, 298 K) of entry **5**, Table 1. The presence of only the peak at 5.27 ppm relating to the olefinic proton of unit 3.4 is observed.


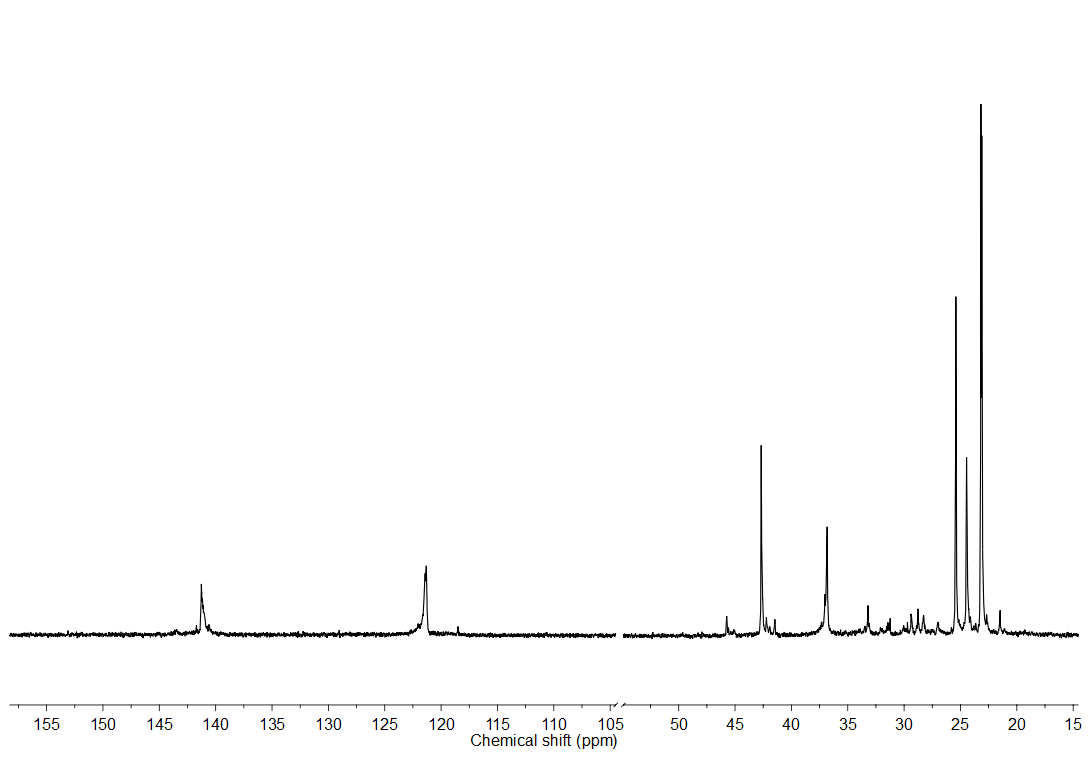


**Figure S5**.^13^C NMR (100 MHz, CDCl_3_, 298 K) of poly(VCH) (entry **2**, Table 1) obtained with catalyst **1.**


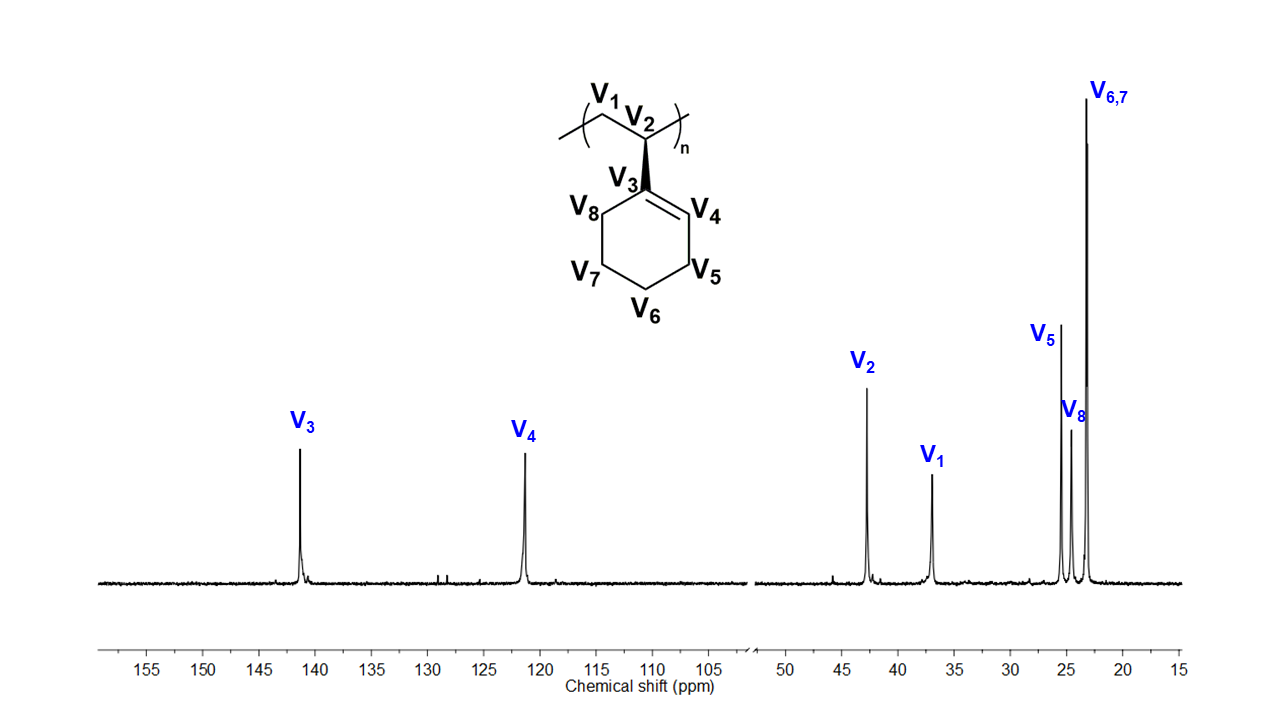


**Figure S6**.^13^C NMR (100 MHz, CDCl_3_, 298 K) of 3,4-isotactic poly(VCH) (entry **5**, Table 1) obtained with catalyst **2.**


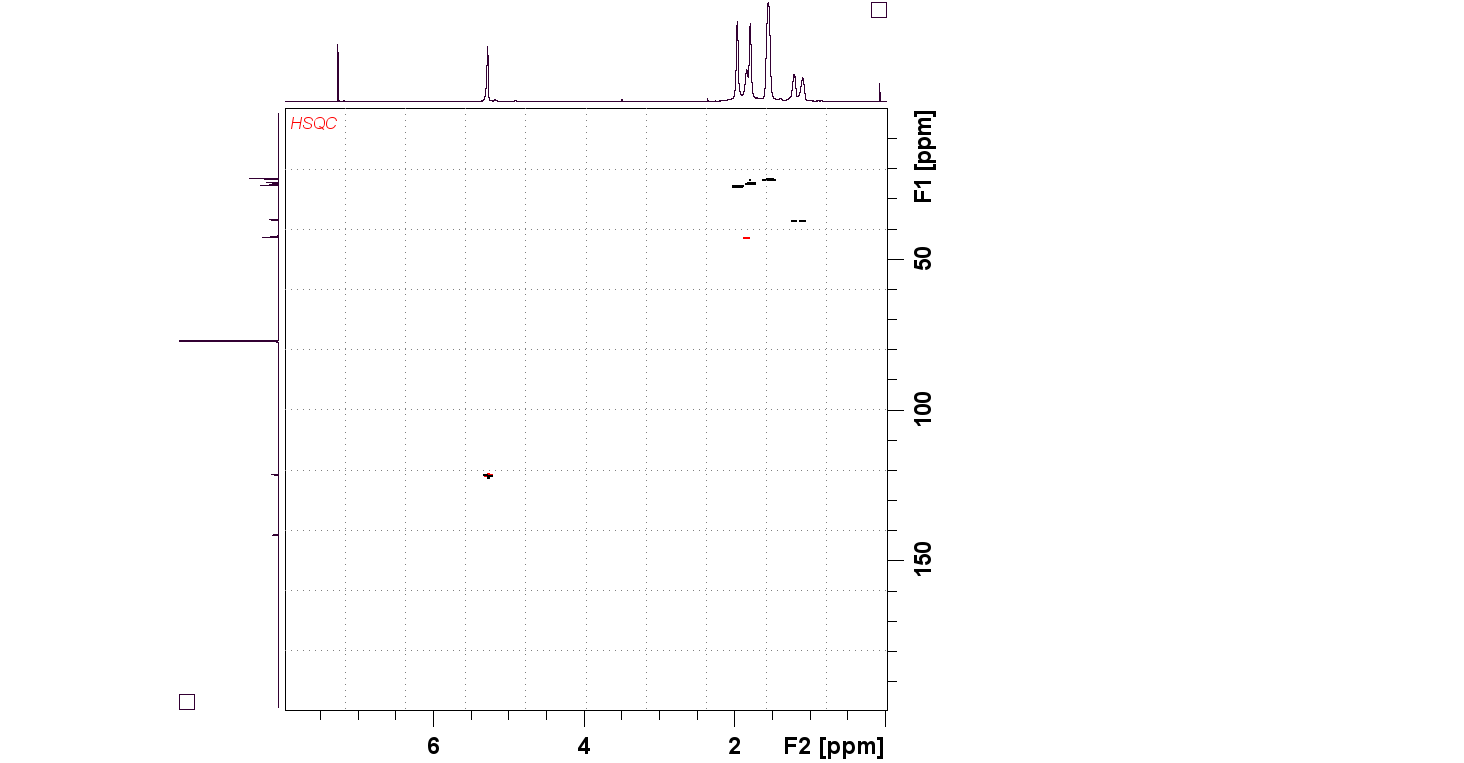


**Figure S7**. HSQC NMR spectrum (600 MHz/100MHz, CDCl_3_, 298 K) of poly(VCH) (entry **8**, Table 1).


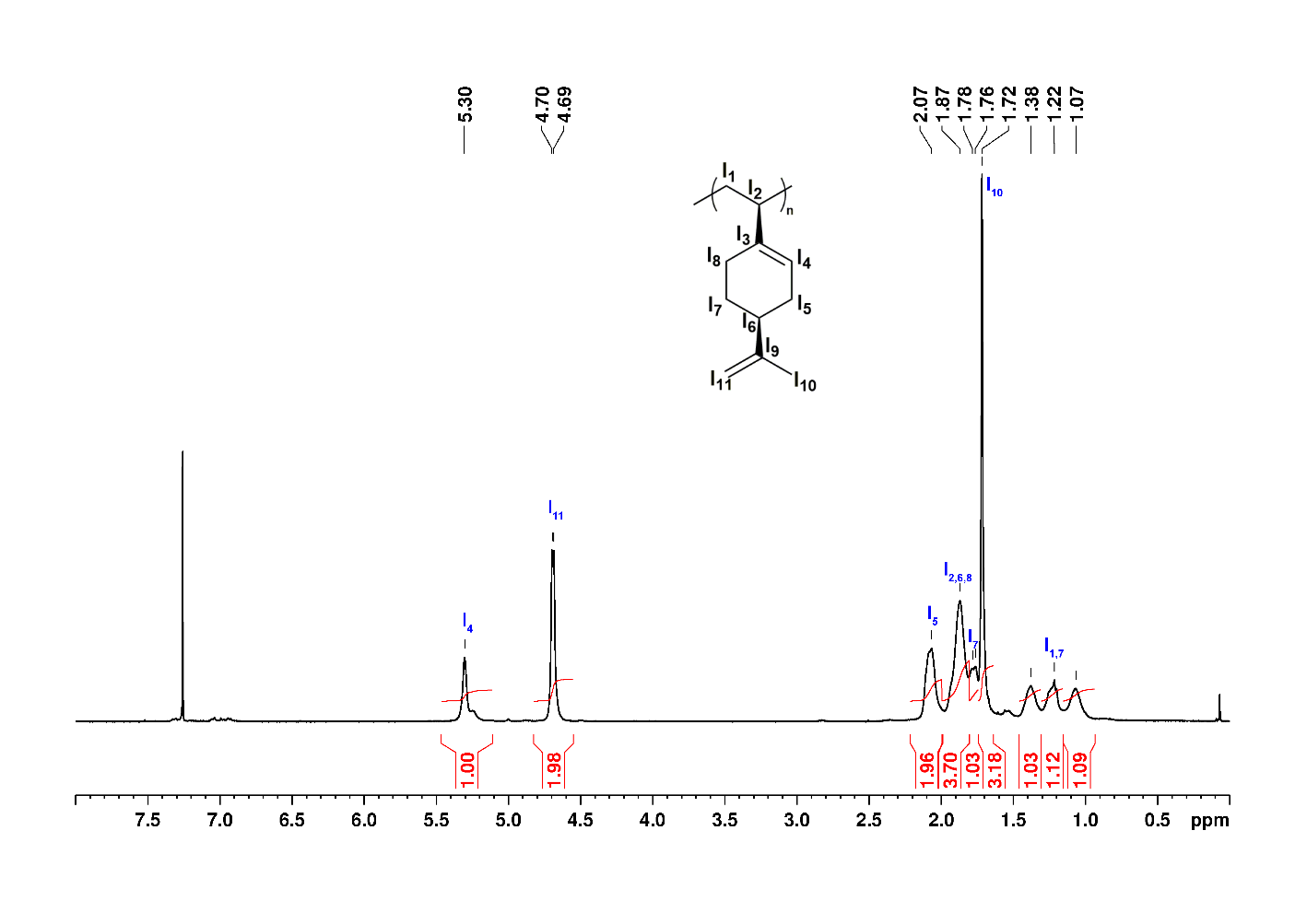


**Figure S8**. ^1^H NMR (300 MHz, CDCl_3_, 298 K) of poly(IVC) from entry **9**, Table 1.


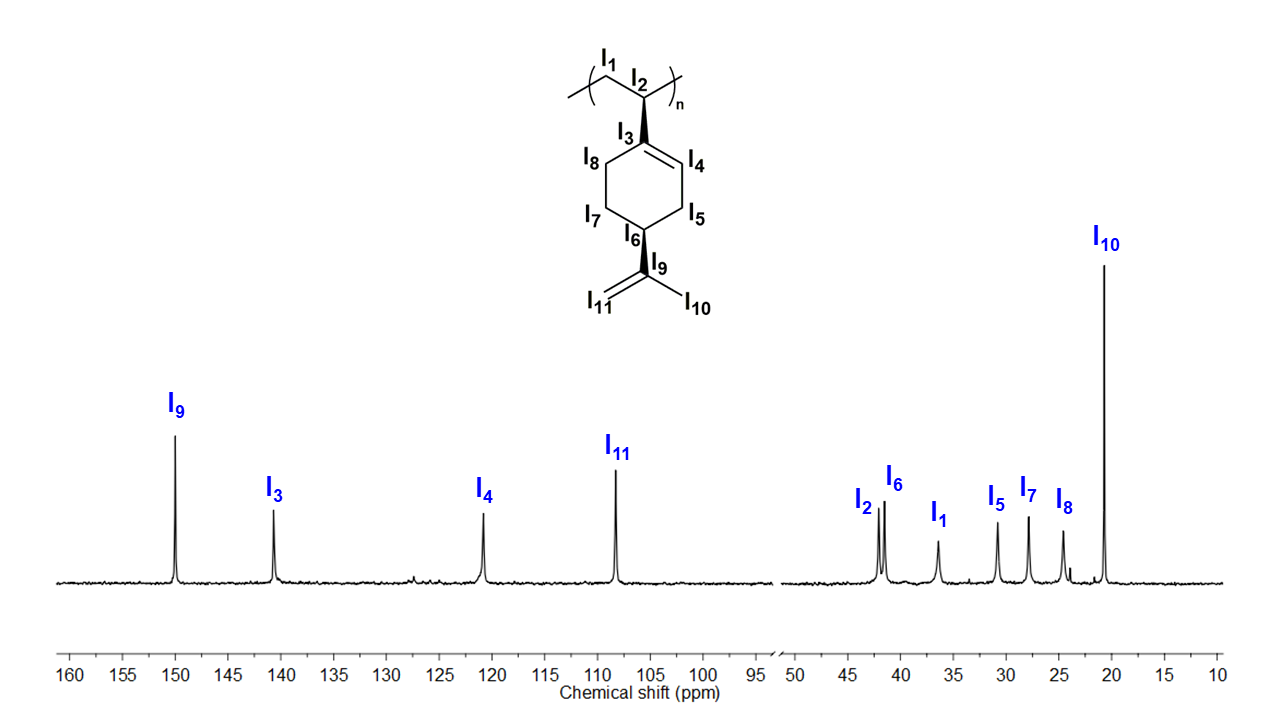


**Figure S9.** ^13^C NMR (100 MHz, CDCl_3_, 298 K) of isotactic poly(IVC) (entry **9**, Table 1) obtained with catalyst **2.**


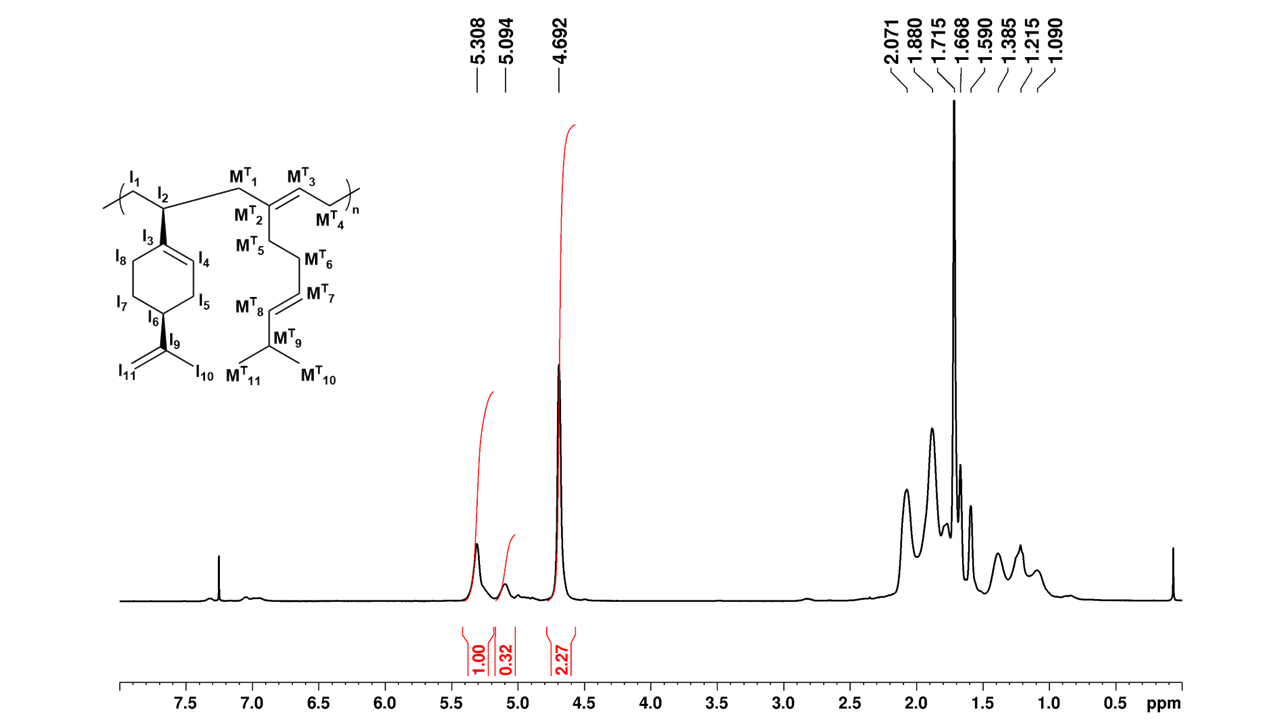


**Figure S10**. ^1^H NMR (300 MHz, CDCl_3_, 298 K) of IVCM copolymer from entry **3**, Table 2.


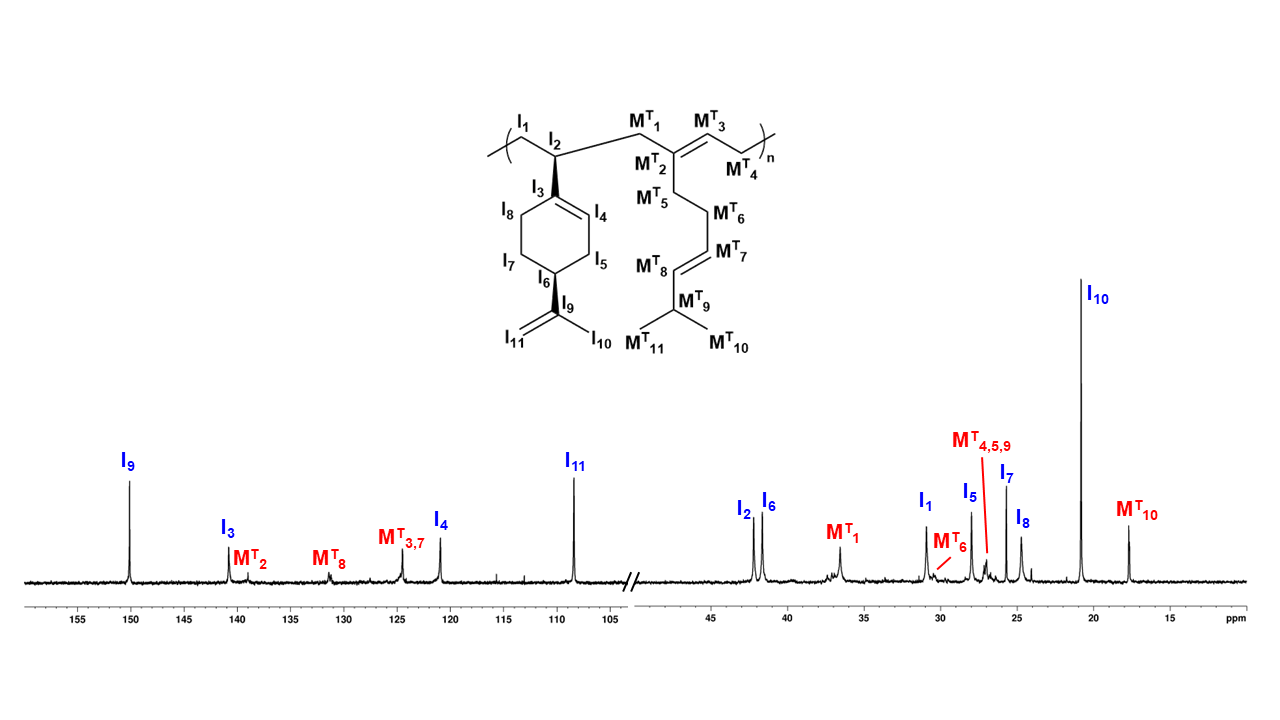


**Figure S11**. ^13^C NMR (100 MHz, CDCl_3_, 298 K) of IVCM copolymer from entry **3**, Table 2.


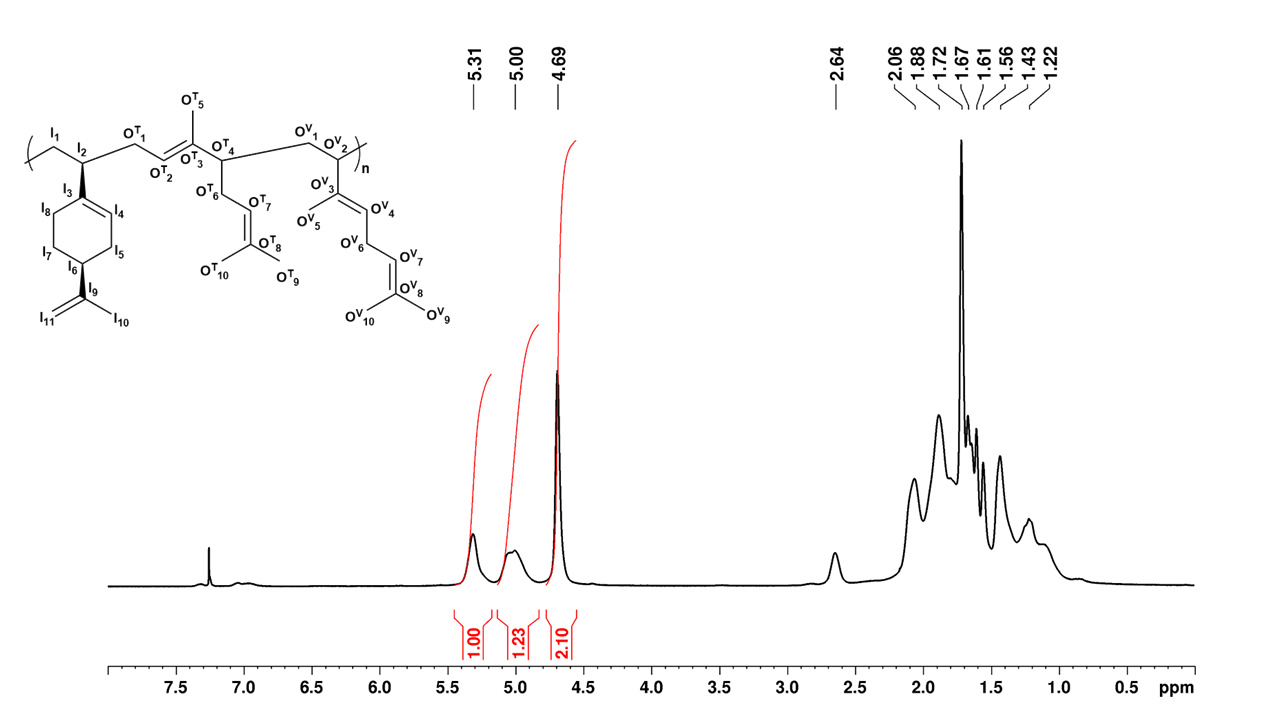


**Figure S12**. ^1^H NMR (300 MHz, CDCl_3_, 298 K) of IVCO copolymer from entry **5**, Table 2.


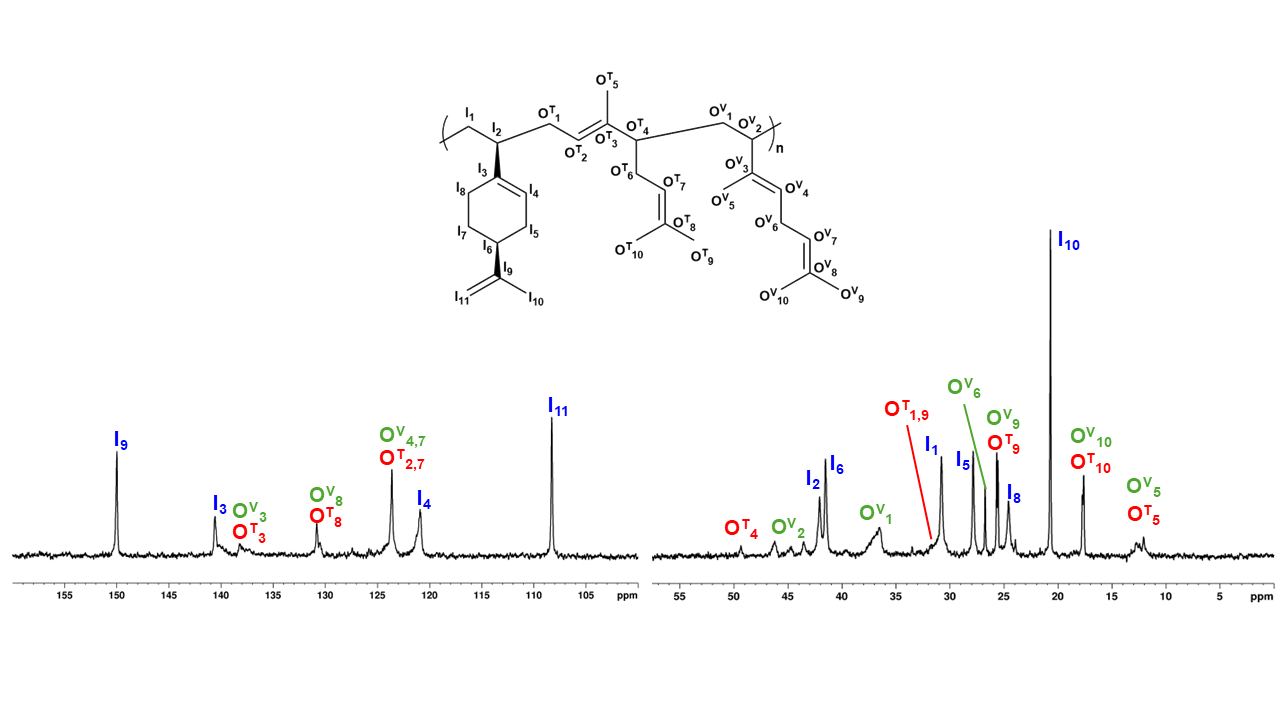


**Figure S13**. ^13^C NMR (100 MHz, CDCl_3_, 298 K) of IVCO copolymer from entry **5**, Table 2.

# **Hydrogenation of poly(vinylcyclohexene)**


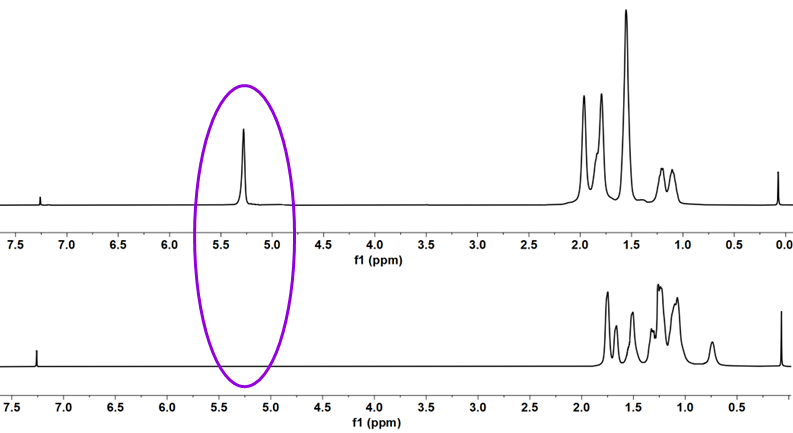


**Figure S14**. ^1^H NMR (400 MHz, CDCl_3_, 298 K) of poly(vinylcyclohexene) (sample **8**, Table 1) before (top) and after (bottom) hydrogenation.

**Figure S15**. DSC thermogram of the hydrogenated polymer (sample **8**, Table 1).

# **DSC Thermal Analyses**


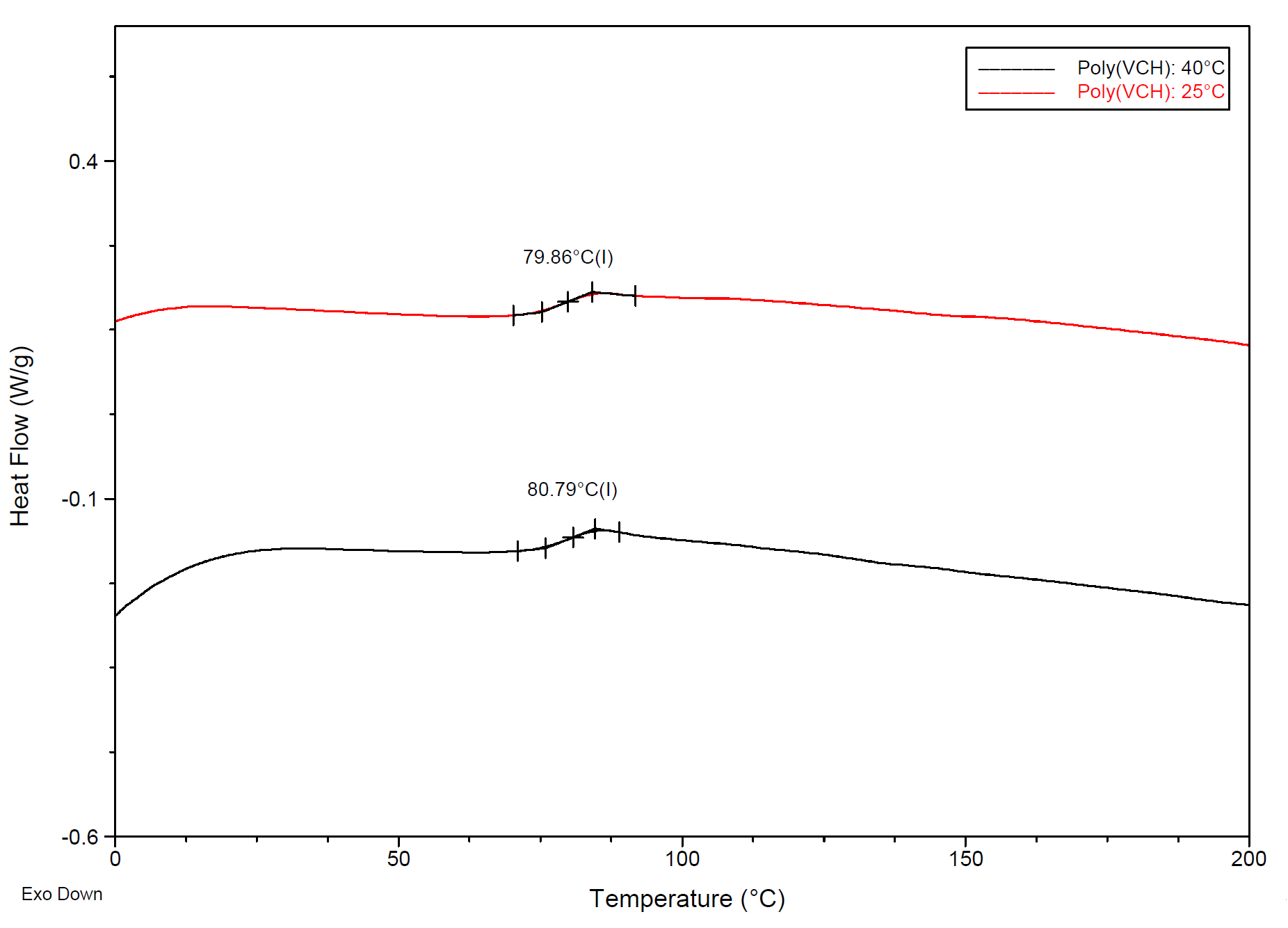


**Figure S16**. DSC thermograms of poly(VCH) obtained with catalyst **1** at different temperatures (see runs **1** and **2**, Table 1).


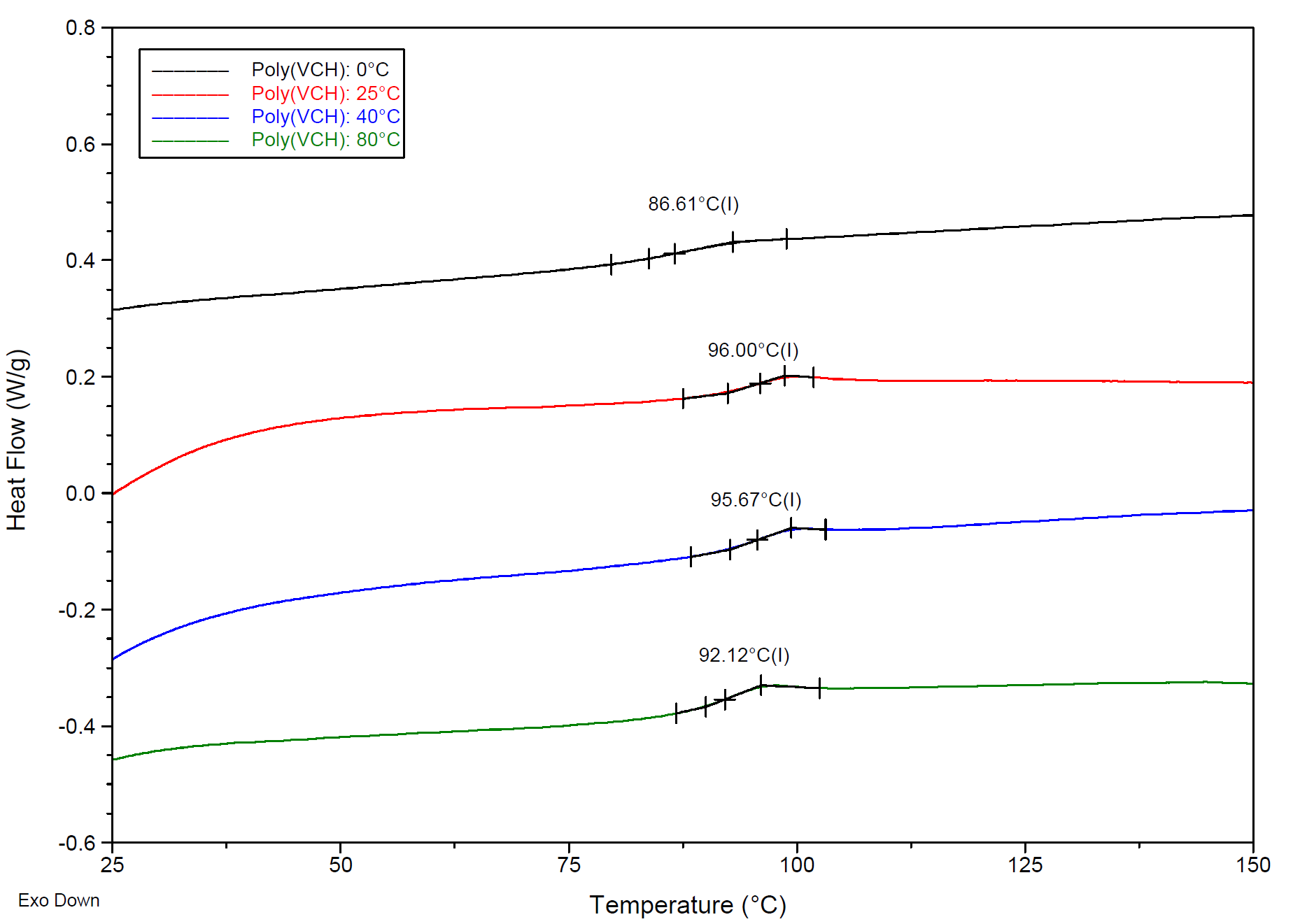


**Figure S17**. DSC thermograms of poly(VCH) obtained with catalyst **2** at different temperatures (see runs **3-6**, Table 1).


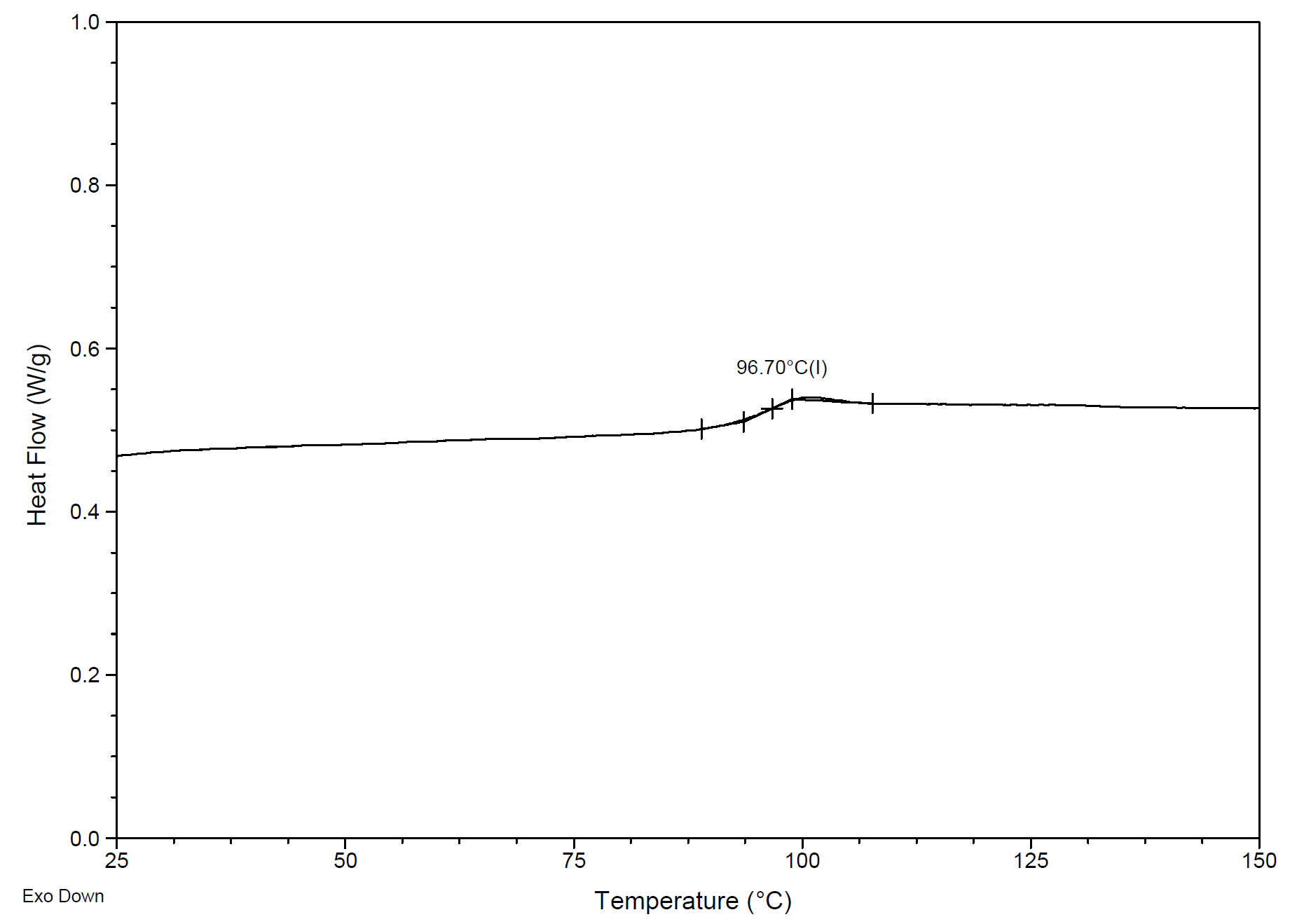


**Figure S18**. DSC thermogram of poly(VCH) from run **7**, Table 1.


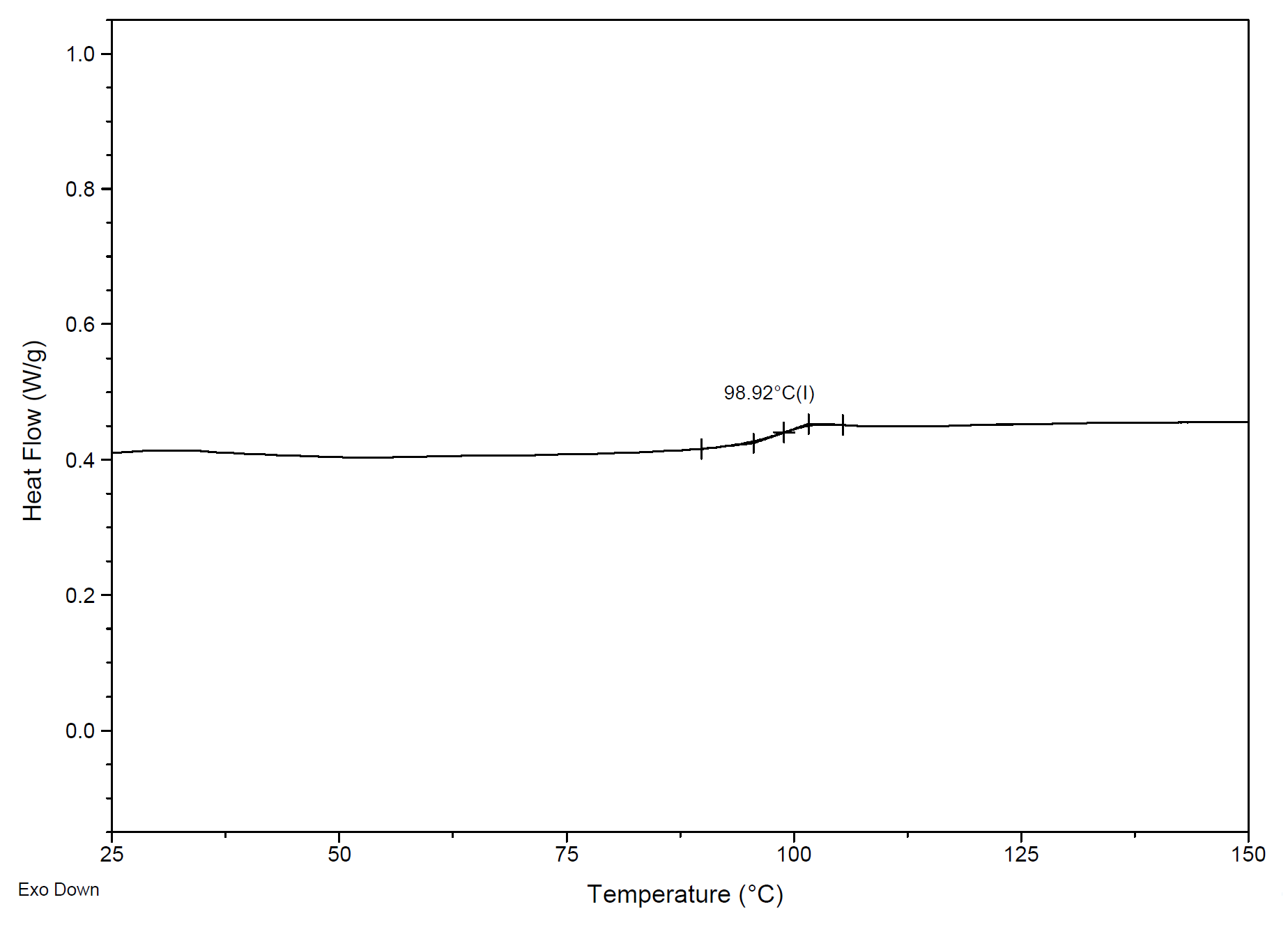


**Figure S19.** DSC thermogram of poly(VCH) from run **8**, Table 1.


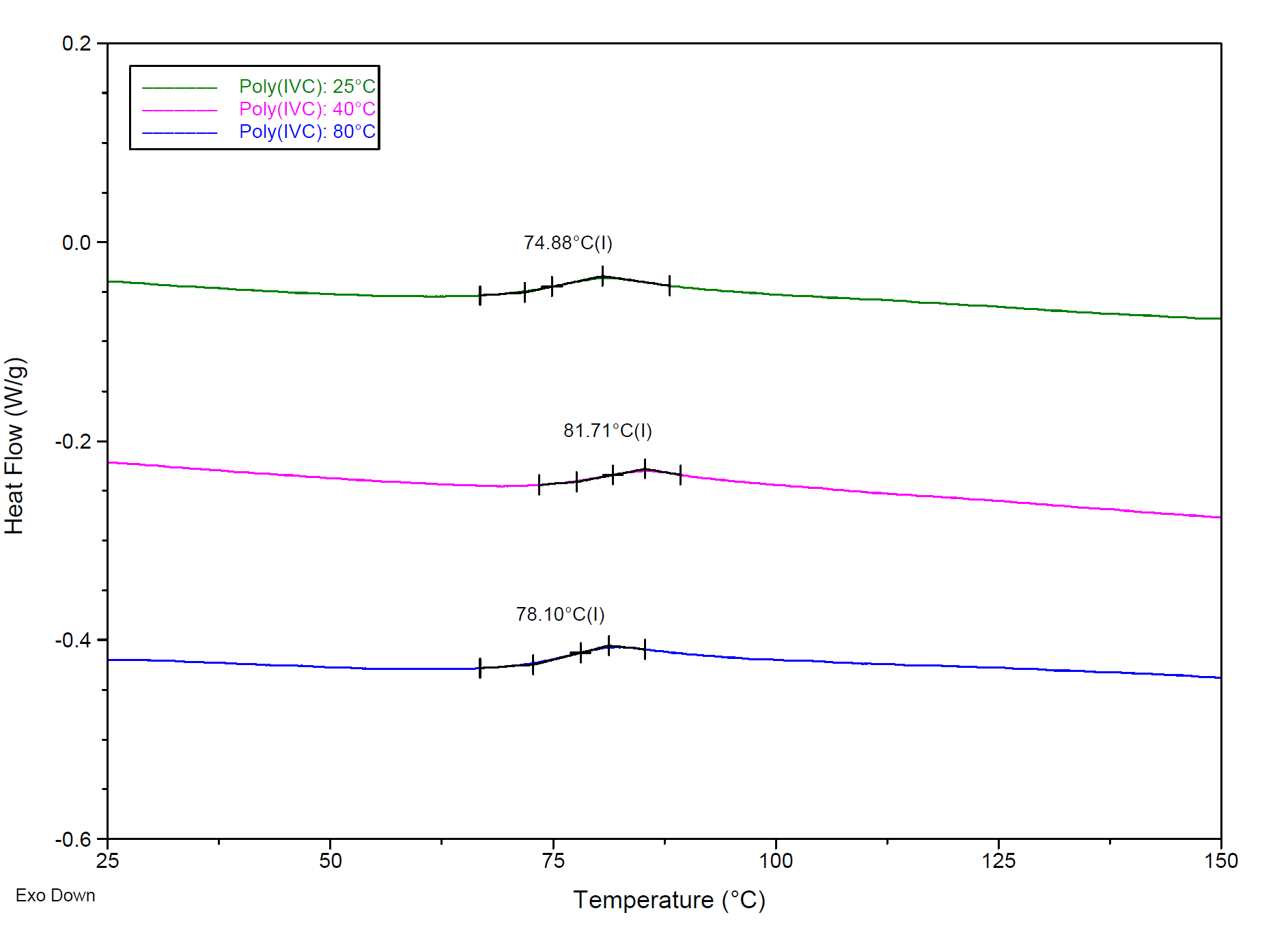


**Figure S20.** DSC thermograms of poly(IVC) obtained with catalyst **2** at different temperatures (see runs **9-11**, Table 1).


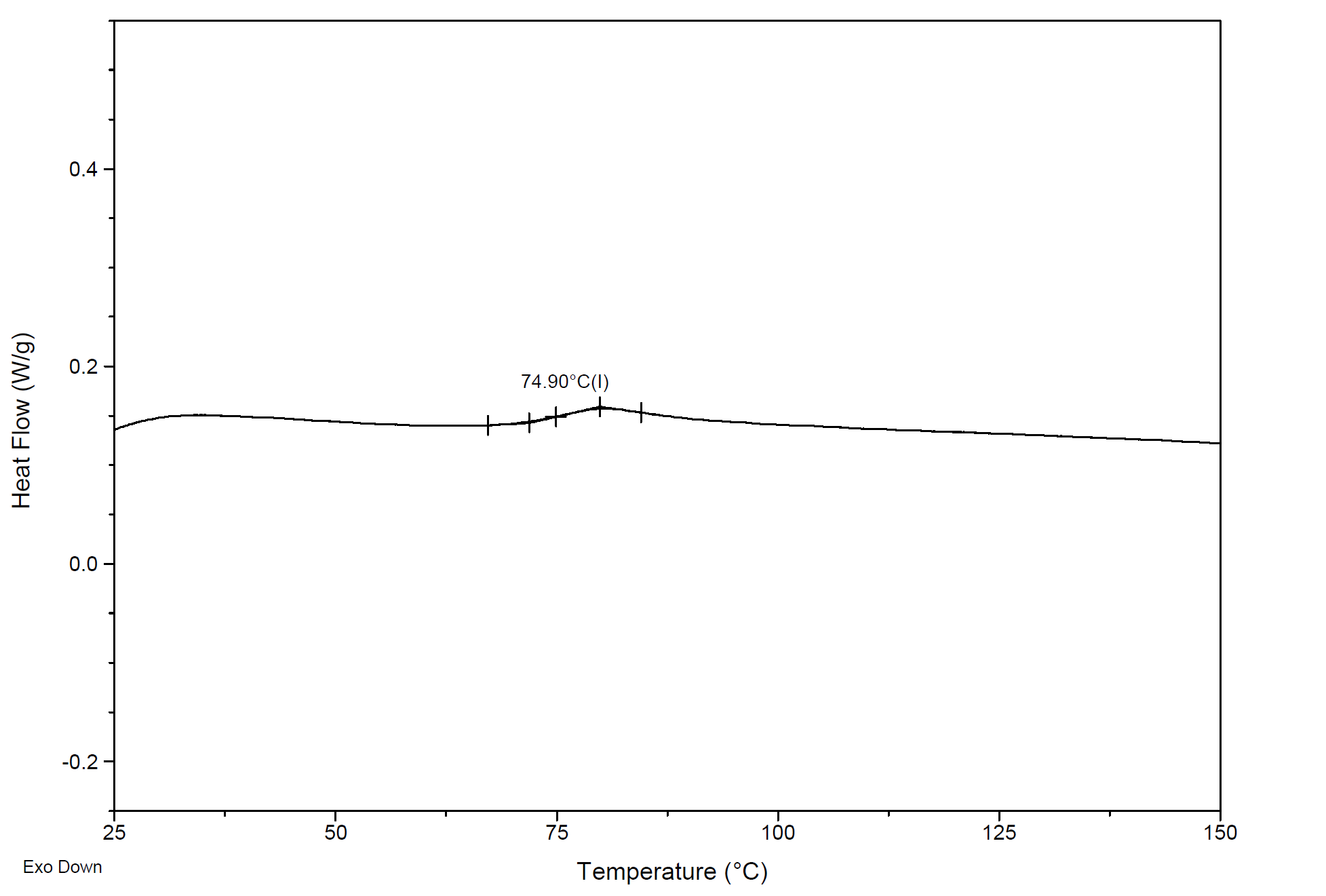


**Figure S21.** DSC thermogram of poly(IVC) from run **12**, Table 1.


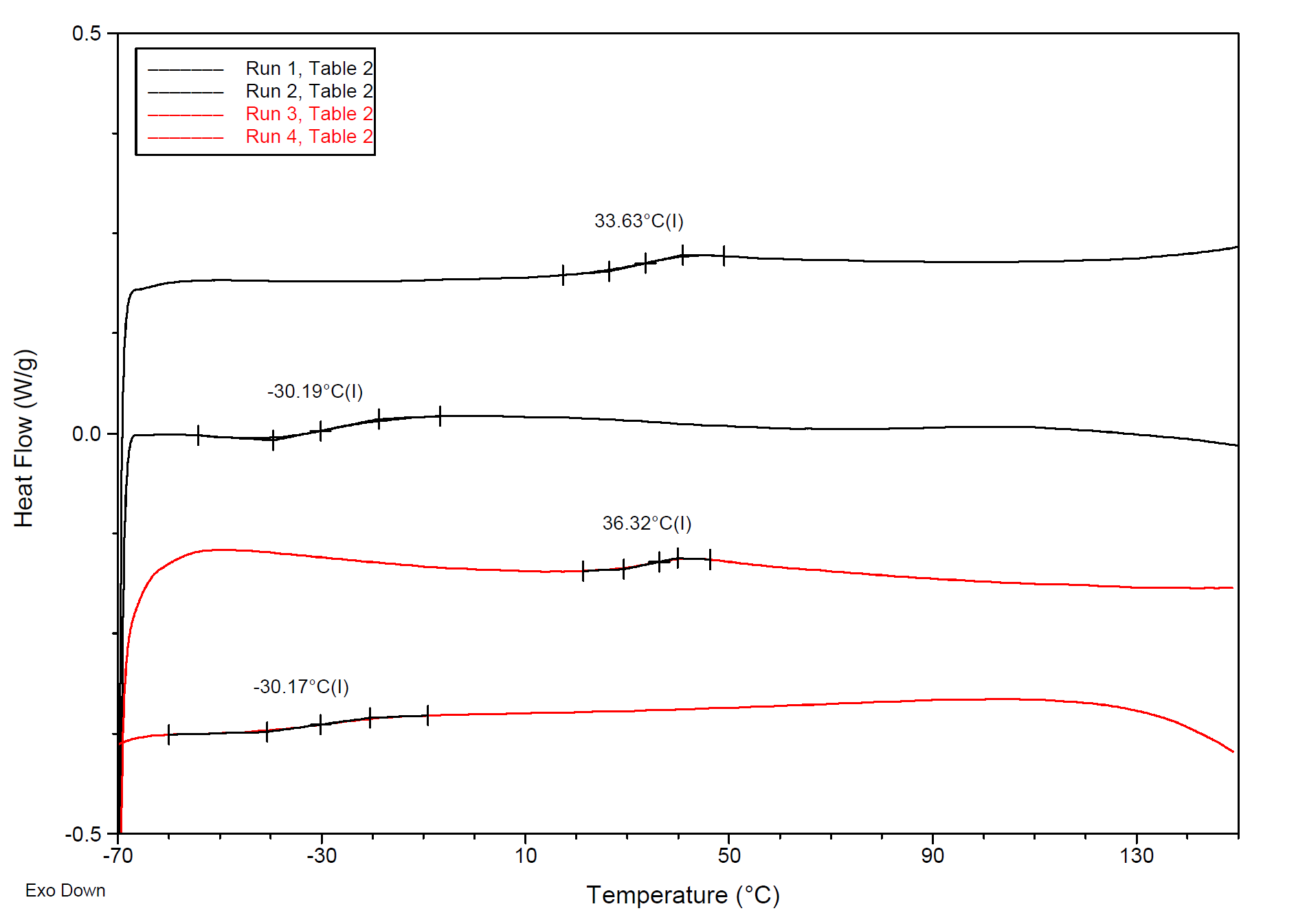


**Figure S22.** DSC thermograms of IVCM copolymers obtained with catalyst **1** (black curves) and catalyst **2** (red curves), see runs **1-4**, Table 2.


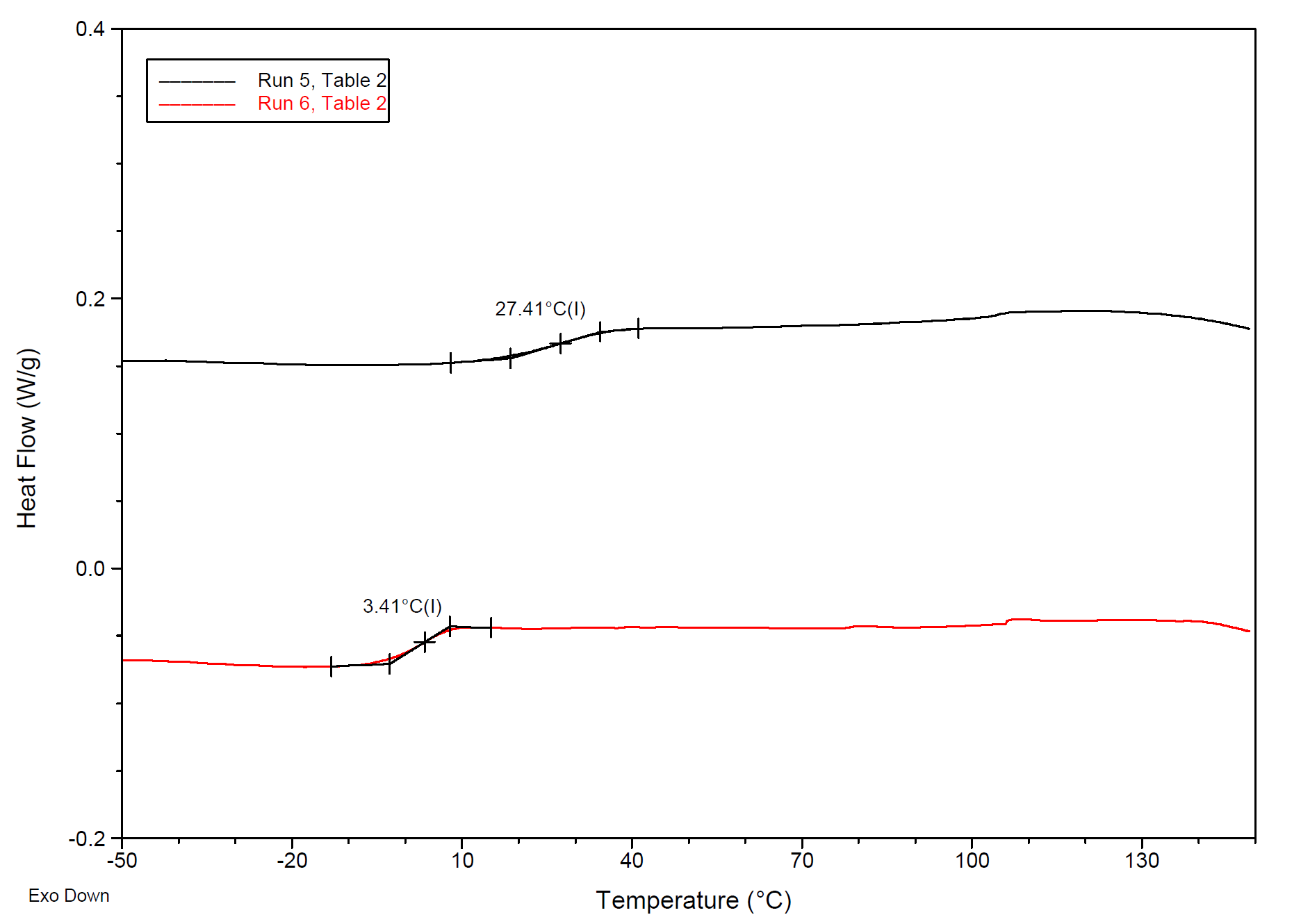


**Figure S23.** DSC thermograms of IVCO copolymers from runs **5** and **6**, Table 2.

# **Thermogravimetric Analyses**


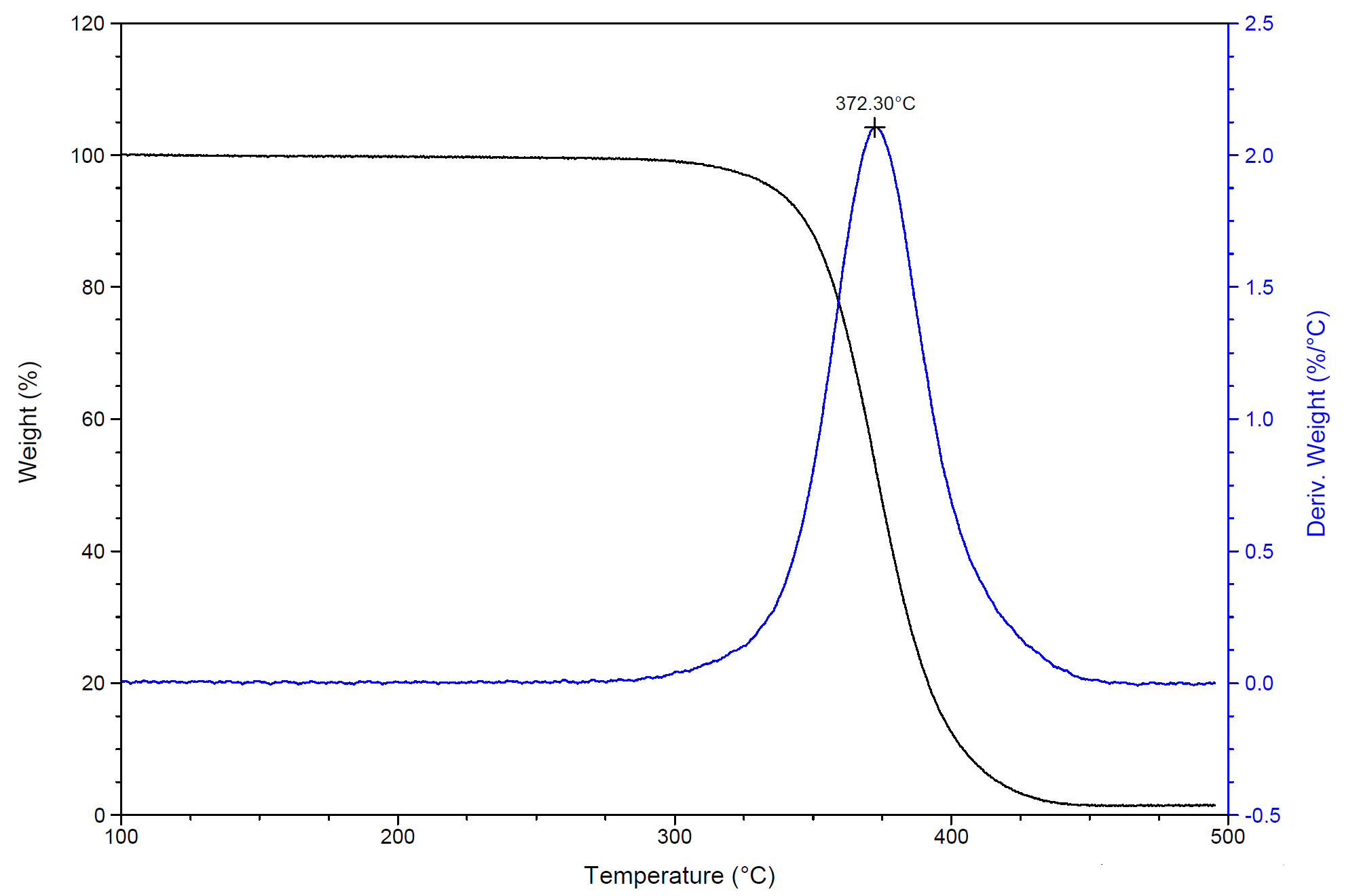


**Figure S24.** TGA traces of the poly(VCH) from run **5**, Table 1.


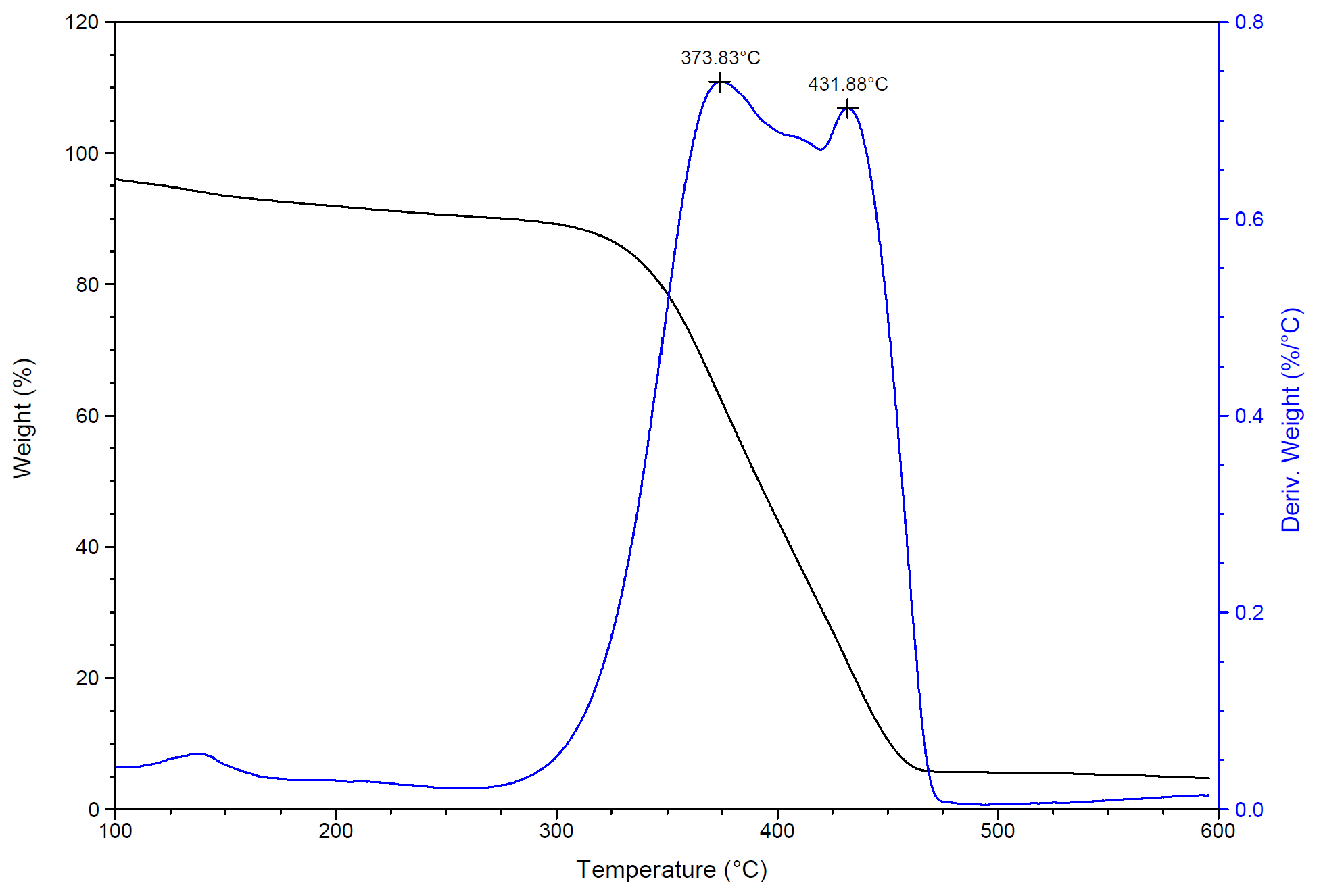


**Figure S25.** TGA traces of the poly(IVC) from run **11**, Table 1.


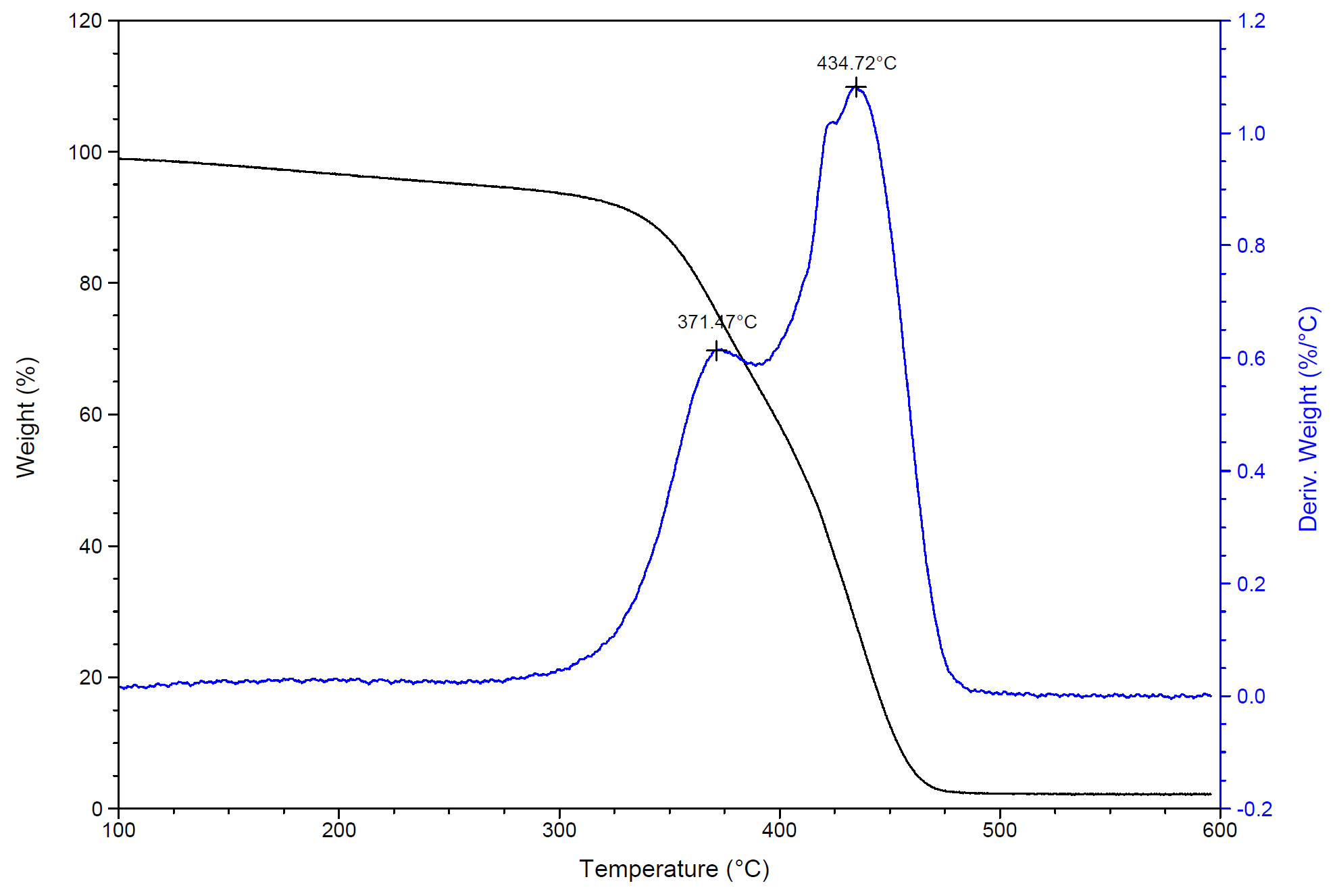


**Figure S26.** TGA traces of the IVCM copolymer from run **1**, Table 2.


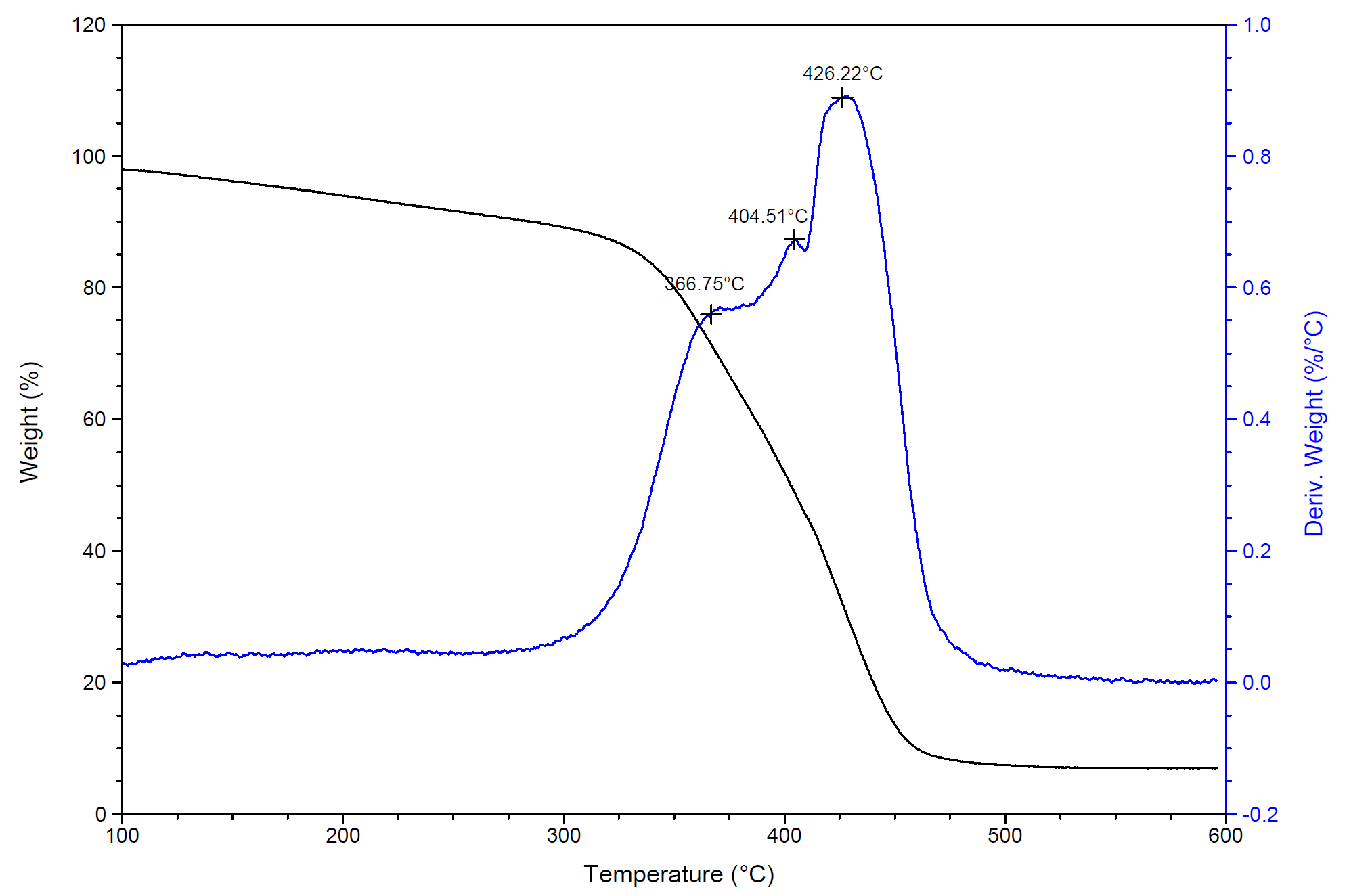


**Figure S27.** TGA traces of the IVCO copolymer from run **5**, Table 2.

# **GPC Analyses**


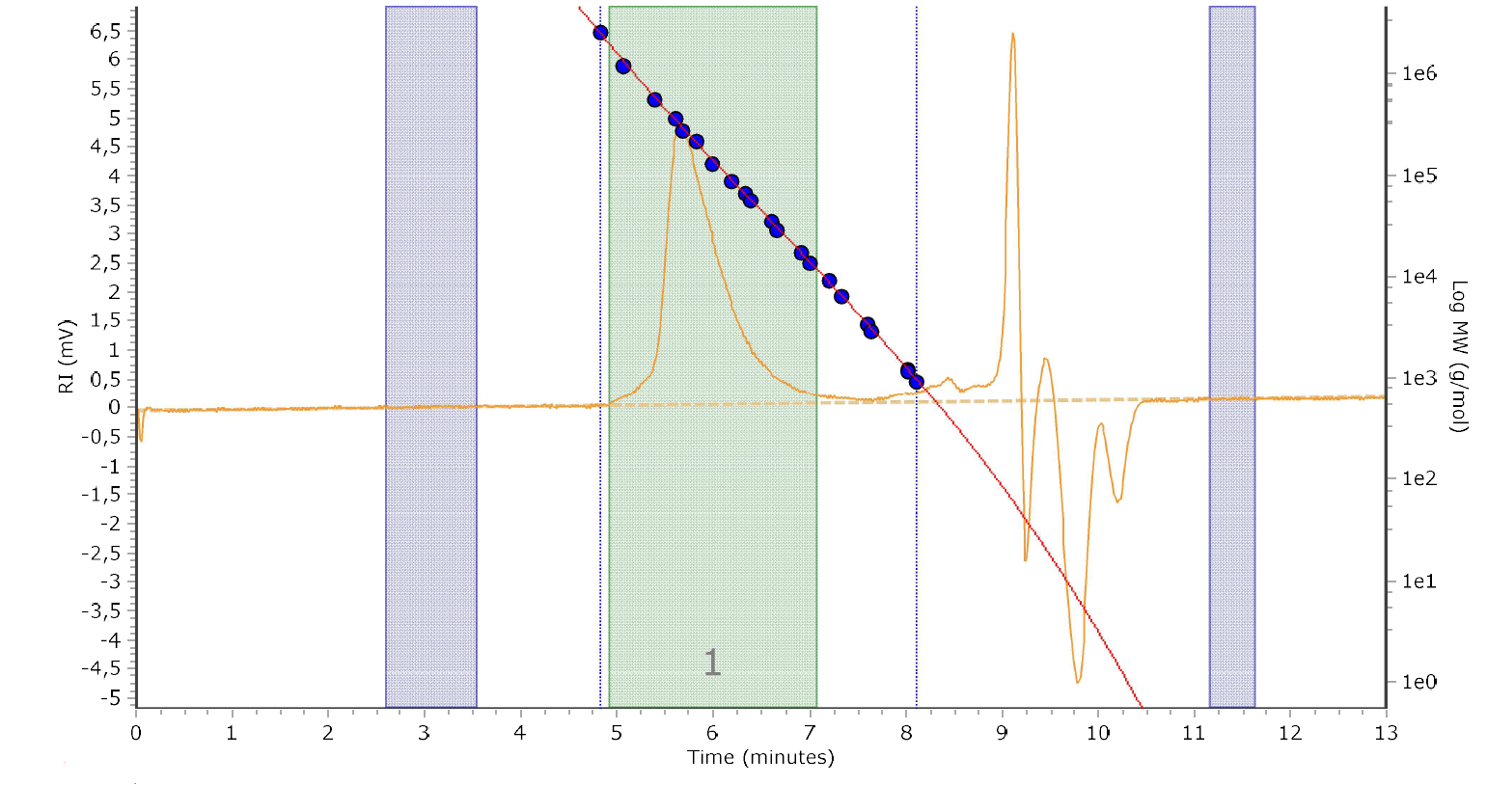

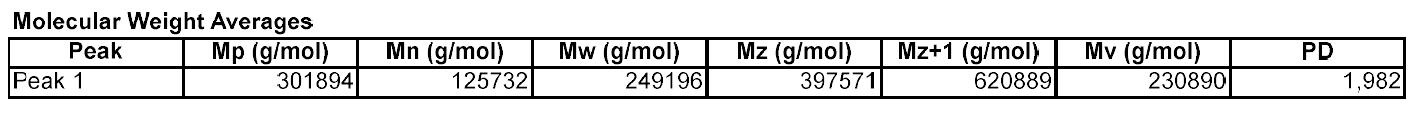


**Figure S28**. GPC curve of poly(VCH) from run **5**, Table 1.


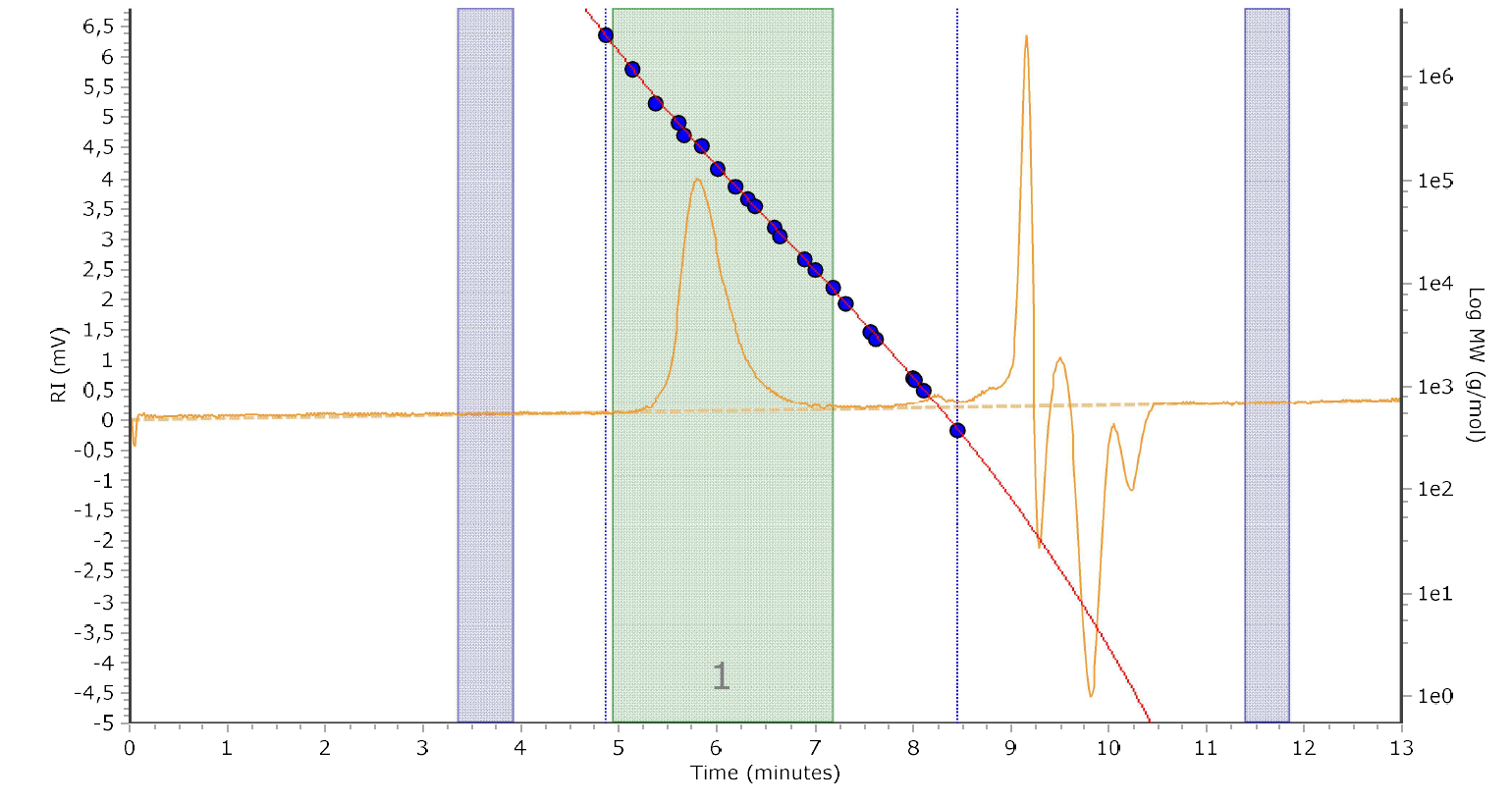


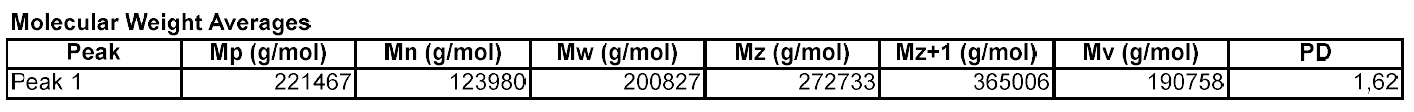


**Figure S29**. GPC curve of poly(IVC) from run **11**, Table 1.


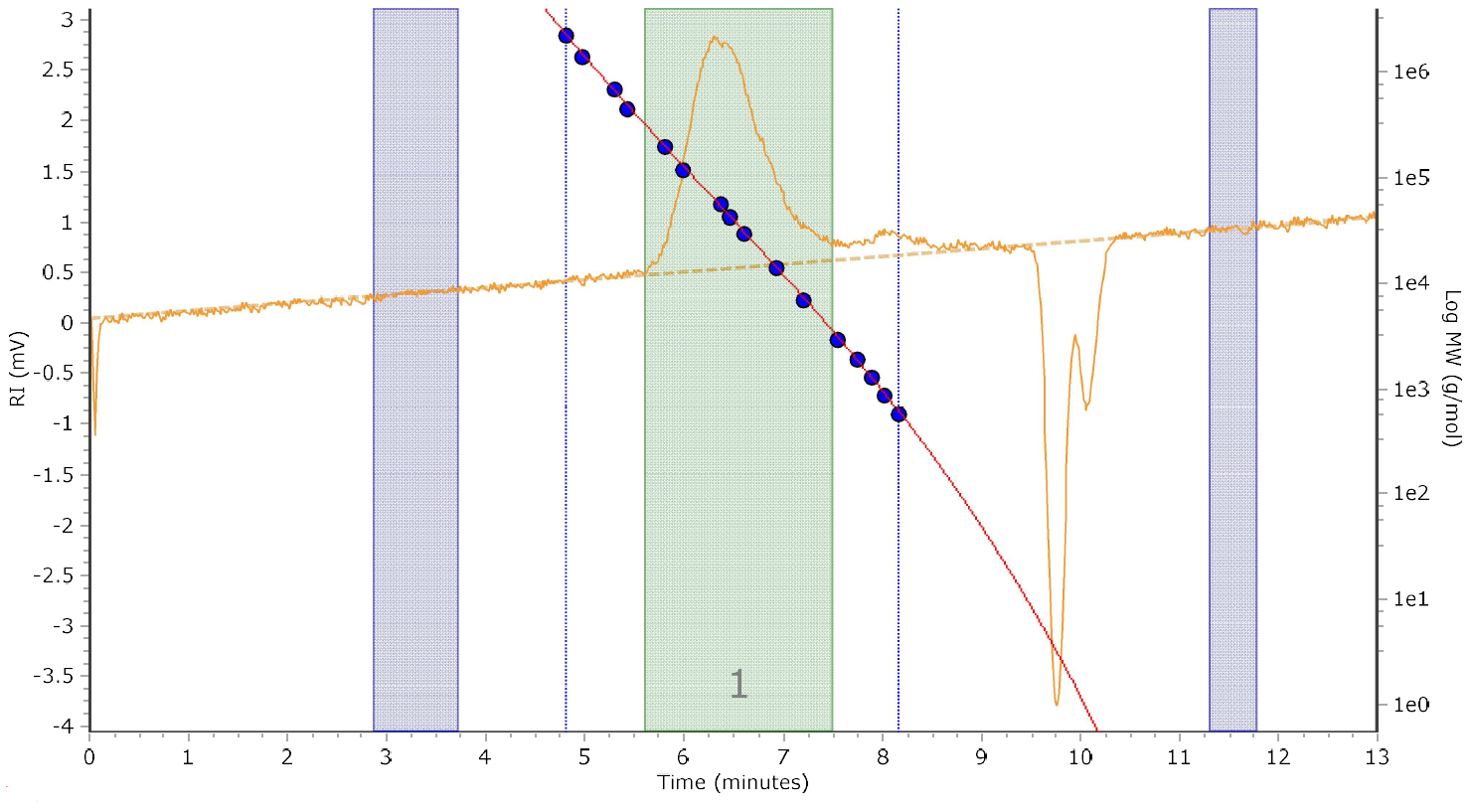


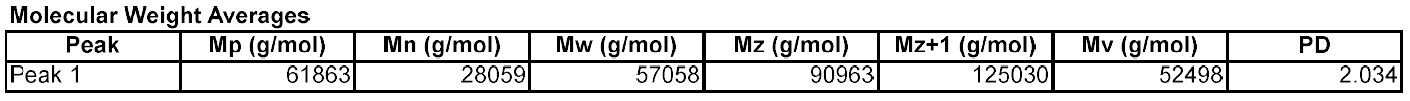


**Figure S30**. GPC curve of IVCM copolymer from run **1**, Table 2.


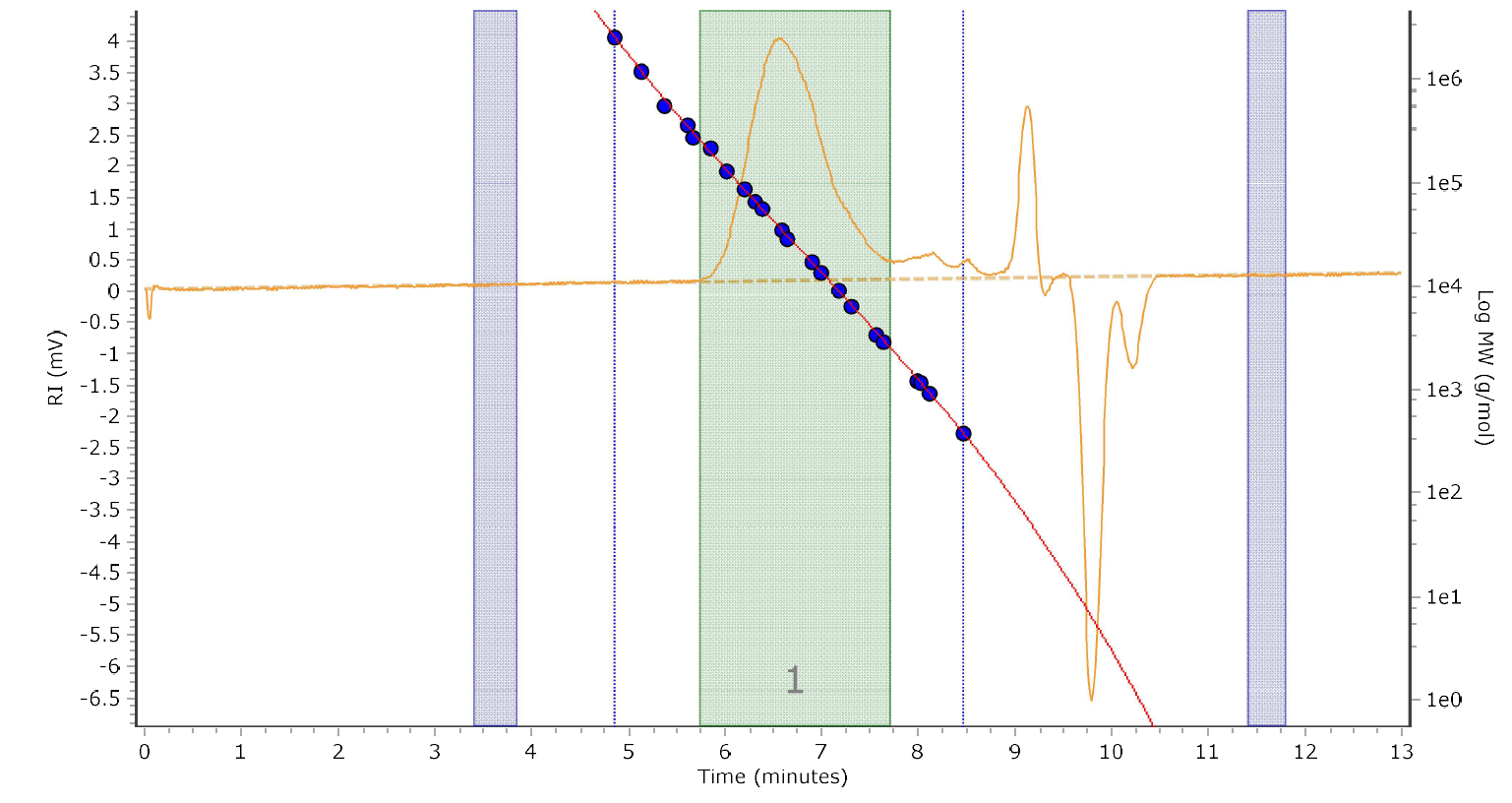


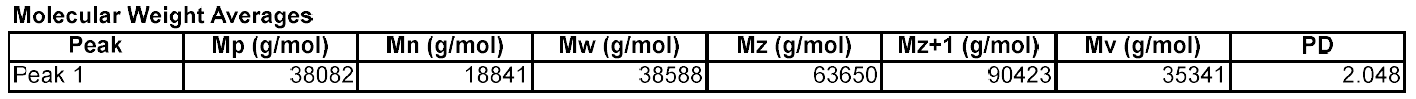


**Figure S31**. GPC curve of IVCM copolymer from run **3**, Table 2.


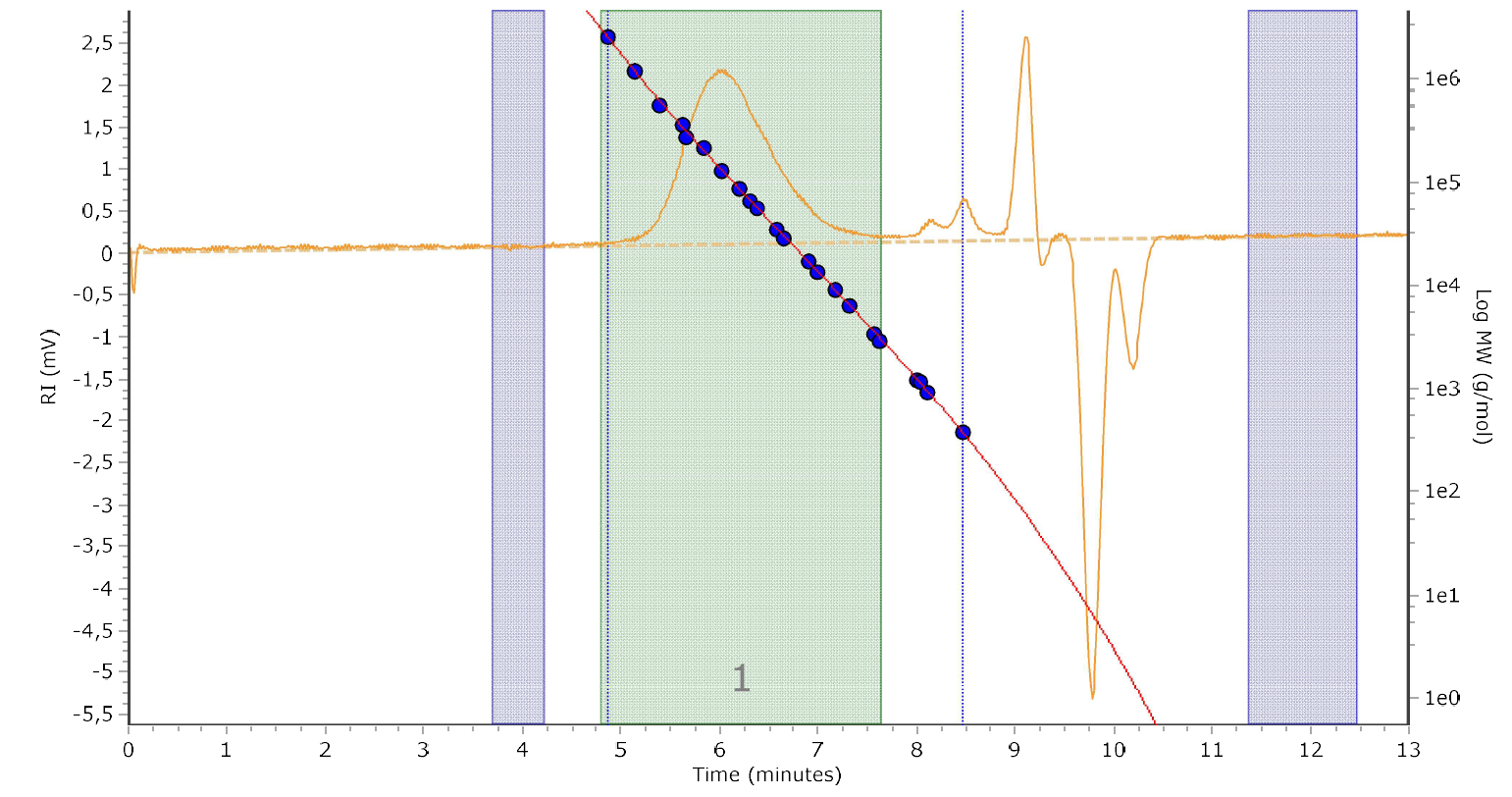


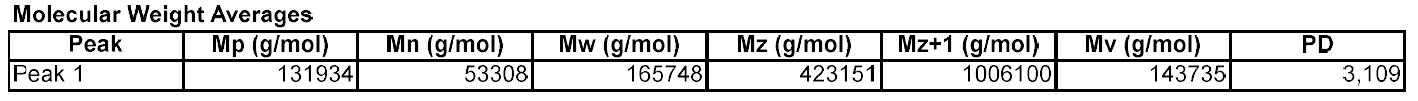


**Figure S32**. GPC curve of IVCO copolymer from run **5**, Table 2.

# **Diffraction Analyses**

**Figure S33.** WAXS profile of as prepared isotactic poly(S-4-isopropenyl-1-vinyl-1-cyclohexene) sample. Similar profiles are obtained for melt pressed and solution cast (from toluene and chloroform) films.

# **Stress-strain curves**

**Figure S34.** Stress-strain curves of isotactic poly(vinylcyclohexene) and isotactic poly(S-4-isopropenyl-1-vinyl-1-cyclohexene) samples.

# **References**

(1) Hahn, C.; Rauschenbach, M.; Frey, H. Merging Styrene and Diene Structures to a Cyclic Diene: Anionic Polymerization of 1‐Vinylcyclohexene (VCH). *Angew Chem Int Ed* **2023**, *62* (28), e202302907. https://doi.org/10.1002/anie.202302907.

(2) Liu, H.; You, F.; Shi, W.; Hu, X.; So, Y.-M.; Shi, X. Rare-Earth-Metal Catalyzed Highly Regio- and Stereoselective Polymerization of Terpene-Derived Conjugated Dienes. *Polymer Chemistry* **2023**, *14* (38), 4474–4480. https://doi.org/10.1039/D3PY00959A.
